# Genomic and Clinical Effects Associated with a Relaxation Response Mind-Body Intervention in Patients with Irritable Bowel Syndrome and Inflammatory Bowel Disease

**DOI:** 10.1371/journal.pone.0123861

**Published:** 2015-04-30

**Authors:** Braden Kuo, Manoj Bhasin, Jolene Jacquart, Matthew A. Scult, Lauren Slipp, Eric Isaac Kagan Riklin, Veronique Lepoutre, Nicole Comosa, Beth-Ann Norton, Allison Dassatti, Jessica Rosenblum, Andrea H. Thurler, Brian C. Surjanhata, Nicole N. Hasheminejad, Leslee Kagan, Ellen Slawsby, Sowmya R. Rao, Eric A. Macklin, Gregory L. Fricchione, Herbert Benson, Towia A. Libermann, Joshua Korzenik, John W. Denninger

**Affiliations:** 1 Gastrointestinal Unit, Massachusetts General Hospital, Boston, Massachusetts, United States of America; 2 Division of Interdisciplinary Medicine & Biotechnology, and Genomics, Proteomics, Bioinformatics and Systems Biology Center, Department of Medicine, Beth Israel Deaconess Medical Center, Boston, Massachusetts, United States of America; 3 Benson-Henry Institute for Mind Body Medicine, Massachusetts General Hospital, Boston, Massachusetts, United States of America; 4 Department of Medicine, Massachusetts General Hospital, Boston, Massachusetts, United States of America; 5 Psychiatry, Massachusetts General Hospital, Boston, Massachusetts, United States of America; 6 MGH Biostatistics Center, Massachusetts General Hospital, Boston, MA, and Harvard Medical School, Boston, Massachusetts, United States of America; 7 Department of Quantitative Health Sciences, University of Massachusetts Medical School, Worcester, Massachusetts, United States of America; 8 Center for Healthcare Organization and Implementation Research (CHOIR), Bedford VA Medical Center, Bedford, Massachusetts, United States of America; Kyushu University Faculty of Medical Science, JAPAN

## Abstract

**Introduction:**

Irritable Bowel Syndrome (IBS) and Inflammatory Bowel Disease (IBD) can profoundly affect quality of life and are influenced by stress and resiliency. The impact of mind-body interventions (MBIs) on IBS and IBD patients has not previously been examined.

**Methods:**

Nineteen IBS and 29 IBD patients were enrolled in a 9-week relaxation response based mind-body group intervention (RR-MBI), focusing on elicitation of the RR and cognitive skill building. Symptom questionnaires and inflammatory markers were assessed pre- and post-intervention, and at short-term follow-up. Peripheral blood transcriptome analysis was performed to identify genomic correlates of the RR-MBI.

**Results:**

Pain Catastrophizing Scale scores improved significantly post-intervention for IBD and at short-term follow-up for IBS and IBD. Trait Anxiety scores, IBS Quality of Life, IBS Symptom Severity Index, and IBD Questionnaire scores improved significantly post-intervention and at short-term follow-up for IBS and IBD, respectively. RR-MBI altered expression of more genes in IBD (1059 genes) than in IBS (119 genes). In IBD, reduced expression of RR-MBI response genes was most significantly linked to inflammatory response, cell growth, proliferation, and oxidative stress-related pathways. In IBS, cell cycle regulation and DNA damage related gene sets were significantly upregulated after RR-MBI. Interactive network analysis of RR-affected pathways identified TNF, AKT and NF-κB as top focus molecules in IBS, while in IBD kinases (e.g. MAPK, P38 MAPK), inflammation (e.g. VEGF-C, NF-κB) and cell cycle and proliferation (e.g. UBC, APP) related genes emerged as top focus molecules.

**Conclusions:**

In this uncontrolled pilot study, participation in an RR-MBI was associated with improvements in disease-specific measures, trait anxiety, and pain catastrophizing in IBS and IBD patients. Moreover, observed gene expression changes suggest that NF-κB is a target focus molecule in both IBS and IBD—and that its regulation may contribute to counteracting the harmful effects of stress in both diseases. Larger, controlled studies are needed to confirm this preliminary finding.

**Trial Registration:**

ClinicalTrials.Gov NCT02136745

## Introduction

Patients with chronic illnesses such as Irritable Bowel Syndrome (IBS) or Inflammatory Bowel Disease (IBD) often have reduced quality of life. IBS is characterized by abdominal pain/discomfort associated with altered bowel function, such as diarrhea or constipation, without gross structural changes or inflammation [1]; IBD is characterized by gross inflammation in the gastrointestinal (GI) tract which can result in symptoms such as abdominal pain, cramping, diarrhea and bloody stools. They are thought to have different underlying pathophysiologic processes, yet both conditions are believed to be influenced by psychosocial factors, in particular stress. Therefore, mind-body interventions (MBIs) that are intended to reduce stress may be promising treatments for these disorders. In the present study, we examine a 9-week relaxation response-based mind-body intervention (RR-MBI) for a mixed group of IBS and IBD patients.

### Promise of mind-body interventions in GI disease

A number of studies have demonstrated the beneficial impact of non-pharmacological therapies for IBS, for example, hypnosis, cognitive behavioral therapy (CBT), and stress management education [2–4]. Studies suggest that patients with IBS benefit more from CBT treatment than from routine medical care alone [3]. Relaxation or multimodal therapies (a combination of relaxation therapy, education and psychotherapy) have also been shown to be beneficial in IBS [3,5–11]. A Cochrane review of the literature prior to 2009 noted that psychological interventions taken together—including CBT, interpersonal psychotherapy and relaxation/stress management—showed symptom score improvements and improvements in quality of life over the short term (i.e., 3-months) [12]; however, the durability of these interventions was less clear [13]. Since 2009, a number of larger clinical trials in IBS using a variety of psychological modalities have confirmed the short-term benefit of both individual and group sessions [4,13–18].

In IBS, studies implicating psychological and psychosocial stressors, heightened stress responsivity, immune abnormalities, distress-amplifying cognitive styles, psychiatric co-morbidity, and nociceptive abnormalities have led to the formulation of multi-level models which hypothesize a dynamic interaction between all of these factors. In IBD, similar studies have suggested that stress may be a risk for IBD flares of all types [19] and that depression may be a predisposing factor for the development of one of the IBD subtypes, Crohn’s disease [20].

Although less work has been done with non-pharmaceutical treatment for IBD, a small number of studies have shown a benefit in IBD as well. According to a Cochrane review (2011) examining the effects of 21 studies using psychotherapy, patient education alone, or multimodal interventions in adult patients with IBD, showed no change on health-related quality of life, coping, emotional state, and disease activity [12]; however, in adolescents there were positive short-term effects of psychotherapy on most outcomes assessed, including quality of life and depression. In addition, a recent randomized controlled trial of three relaxation-training sessions and at home practice of guided imagery compared to a wait-list control showed improvements in quality of life and mood, as well as decreases in pain and stress in IBD patients [21].

While there are clear pathophysiological differences between IBS and IBD, in both disorders chronic pain and negative impacts on psychological well-being can cause patients significant stress; as a result, treatment for neuropathic pain, anxiety, and depression may be common targets in both IBS and IBD. These are areas where psychological interventions such as MBIs may have an impact on both disorders.

### The Relaxation Response-Based Mind Body Intervention

The foundation of the RR-MBI used in this trial is elicitation of the relaxation response (RR), which has been described as a “wakeful hypometabolic” state [22] found to be effective in combating the negative impacts of the stress response, described by Cannon [23] as the “fight-or-flight” response. The RR is a physiological state characterized by decreased arousal of the sympathetic nervous system [22], and can be elicited using many different techniques, including breath awareness, guided imagery, meditation, and yoga. RR-MBIs have been shown to reduce medical symptoms of patients who suffer from chronic medical illnesses [24], facilitate the elimination of antihypertensive medication among patients with high blood pressure [25], and lower the risk factors for cardiac events among participants of a cardiac rehabilitation program [26]. In addition, RR-MBIs appear to be effective in groups made up of patients with many different symptoms and diagnoses [24].

Although MBIs have been investigated and used clinically for many years, their biological mechanisms of action are just beginning to come to light. A pair of studies by our group examined differential gene expression in a group of mind-body practice naive participants before and after 8-weeks of RR-elicitation training (short-term comparison), as well as in naive participants before RR-elicitation training compared to a matched group of long-term practitioners of mind-body techniques (long-term comparison) [27,28]. Dusek et al. (2008) found that more than 3,000 genes were differentially expressed in the long-term and short-term comparisons. Using gene set enrichment and interactive network analysis of RR-affected pathways, Bhasin et al. (2013) found that decreased expression of the NF-κB gene, along with its upstream and downstream targets, may be central to beneficial effects of RR elicitation [27]. Other interventions targeting stress (e.g., mindfulness-based stress reduction and cognitive-behavioral stress management) have been shown to have similar effects on NF-κB and other pro-inflammatory molecules [29,30]. Such findings, while requiring confirmation in additional studies, are provocative and suggest genomic changes caused by RR-eliciting practices counter-regulate genes induced by stress and inflammation.

While RR-MBIs elicit beneficial effects in a variety of medical illnesses, they have not been tested with IBS and IBD patients. As summarized above, there is good evidence to suggest that MBIs, like other psychological interventions, may be helpful in IBS. Furthermore, while prior work has not shown strong evidence for beneficial effects of psychological interventions in IBD, our recent genomics work demonstrating genetic expression changes in inflammatory pathways in response to an RR-MBI [27], suggests a mechanism through which MBIs might be beneficial in IBD. In this pilot study, we examined whether an RR-MBI could be effectively delivered to mixed groups of IBS and IBD patients and determined the effects of the intervention on quality of life, inflammatory markers, and gene expression using transcriptional profiling.

## Methods

### Study design

This pilot, single center, single-arm, open-label study utilized an RR-MBI for the treatment of IBS and IBD using elicitation of the RR through meditation techniques and cognitive skill building, as established by the Benson-Henry Institute for Mind Body Medicine at the Massachusetts General Hospital (BHI). The protocol for this trial and supporting TREND checklist are available as supporting information; see S1 TREND Checklist and S1 Protocol. All study procedures were approved by the Partners Healthcare/Massachusetts General Hospital Institutional Review Board. The study was not initially registered on ClinicalTrials.gov given that it was not a randomized clinical trial (and registration was not required for such studies when it was initiated in 2009); however, because of changes in registration requirements, the study was subsequently registered on ClinicalTrials.gov. The authors confirm that all ongoing and related trials for this intervention are registered.

### Participants

Patients with documented IBS (confirmed by the Rome III diagnostic criteria for 6 months) or IBD (upper limit for HBI [Harvey-Bradshaw index] = 20, upper limit of SCCAI [simple clinical colitis activity index] = 18) by their primary care provider or gastroenterologist were eligible to participate. Patients with IBS were excluded if they had abdominal surgery in the past 5 years (with the exception of appendectomy, cholecystectomy) or documentation of GI motility disorder. Patients with IBD were excluded if they used NSAIDS chronically, were on a prednisone dose ≥20 mg/day, or if surgery was anticipated in the 10-weeks following enrollment. Patients were also excluded if they had current evidence of duodenal ulcer, gastric ulcer, diverticulitis, esophagitis or infectious gastroenteritis, or any acute gastrointestinal process, as well as if concurrent total parental nutrition or tube feeding were being used. Patients with recent (within the last 4-weeks) changes in IBS/IBD medications, planned changes in diet, or current use of steroids were excluded.

Eligible patients were 18–75 years old and fluent in English. Patients who were pregnant or attempting to become pregnant were excluded. Patients were excluded if they were currently (>3-weeks) practicing Tai Chi, meditation, yoga, individual mind/body based psychotherapy or counseling, as well as if they had initiated psychotherapy within the last 8-weeks, used psychotropic medications (except at stable doses for at least 12-weeks), or had an untreated psychiatric disorder.

Patients were recruited from the Massachusetts General Hospital (MGH) Gastrointestinal Clinic and from a network of primary care providers at via provider referral, self-referral, and advertisements posted in waiting areas starting in June, 2009. Potential participants were pre-screened over the phone by a research nurse and were scheduled for a screening visit, where written informed consent was obtained and interviews were conducted by a psychologist to determine eligibility. Participants continued to receive standard care related to their IBS/IBD during participation in the study.

### Intervention

The RR-MBI involved a 9-week group program conducted by a nurse practitioner or psychologist skilled in MBI, which included a GI-specific session conducted by a physician. The groups met once weekly for 1.5 hours. Three consecutive, mixed-disorder groups, ranging in size from 10 to 18 participants each, were included in the study, conducted between 9/23/2009 and 5/31/2011 with follow-up completion by 6/30/2011. The program was multidimensional and included daily elicitation of the RR using a variety of methods (including breath focus, single-pointed focus, imagery, contemplation, yoga, and mindful awareness); cognitive reappraisal skills, health enhancing behaviors, and the promotion of optimism and acceptance. As described in Table 1, sessions 1–4 focused on developing an understanding of stress physiology and the physiology of the RR, its relationship to the digestive system, and developing a regular practice of eliciting the RR. Sessions 5–9 included information on lifestyle behaviors and the development of cognitive skills to cope with stress. Throughout the course of treatment, participants were asked to elicit the RR at home each day for 15–20 minutes.

**Table 1:** The syllabus for the relaxation response-based group mind body intervention (RR-MBI) for irritable bowel syndrome or inflammatory bowel disease.

Session	Content
Session 1	Overview of Mind-Body Medicine / The Stress Response & The RR
Session 2	Diaphragmatic Breathing / Mini-Relaxation Exercises / Appreciation
Session 3	Mindfulness / Self-Care (Pacing Activities)
Session 4	Introduction to Cognitive Skills / Lifestyle Behaviors: Nutrition
Session 5	Cognitive Restructuring / Cognitive Distortions / Lifestyle Behaviors: Exercise
Session 6	GI Physician Lecture and Q & A
Session 7	Cognitive Skills / RR for Insight / Introduce Journaling Exercise
Session 8	Problem Solving and Communication Skills / Yoga
Session 9	Relapse Prevention

Session content varied by week but always included an activity that elicited the relaxation response (RR) and a discussion of the homework from the previous week. Homework for the sessions included eliciting the RR daily, daily mini-relaxation exercises, filling out a diary sheet, making an entry in an appreciation journal, and practicing cognitive restructuring exercises and other skills previously learned.

### Measures

Participants were assessed at baseline and weeks 5 (mid-intervention), 10 (post-intervention), and 13 (short-term follow-up).

#### Self-Report Measures

To measure symptoms common to IBS and IBD, the State-Trait Anxiety Inventory (STAI-Y), the Pain Catastrophizing Scale (PCS), and the Brief Pain Inventory (BPI) were administered at each assessment. The STAI-Y is a widely used instrument for measuring anxiety in adults. It differentiates between the temporary condition of “state anxiety” and the more general and long-standing quality of “trait anxiety” [31]. The PCS is widely used to assess cognitive and affective responses to pain and to evaluate pain management program outcomes [32]. The BPI allows patients to rate the severity of their pain (BPI-S) and the degree to which their pain interferes with common dimensions of feeling and function (BPI-I) [33].

At each assessment, participants were also administered validated disease-specific instruments—the IBS Quality of Life (IBS-QOL), the IBS Symptom Severity Index (IBS-SSI), and the IBD Questionnaire (IBD-Q)—to assess the disease-specific impact of the intervention. The IBS-QOL is a self-reported quality of life (QOL) measure containing 34 questions specific to IBS that is used to assess the impact of IBS and its treatment on QOL [34]. The IBS Symptom Severity Index (SSI) is a widely used questionnaire measuring IBS-related pain frequency, severity of pain, bloating, bowel habit dissatisfaction and interference with daily life and extra-colonic symptoms on a visual analogue scale [35]. The IBD-Q is a 32-question instrument designed to measure the effects of inflammatory bowel disease on daily function and quality of life [36]. All information from this study have been deposited in the NIH Gene Expression Omnibus database at www.ncbi.nlm.nih.gov/geo under the accession number GSE66824.

#### Biological Samples

Blood was collected at baseline and week-10 for erythrocyte sedimentation rate (ESR) and C-reactive protein (CRP) assays as measures of inflammation and in PAXgene (Qiagen) tubes for transcriptional expression profiling.

### Transcriptional profiling

Total RNA was isolated from whole blood samples collected in PAXgene tubes as described previously [28]. The peripheral blood gene expression profile was assessed on IBS and IBD patients with paired pre- and post-intervention samples using HT U133 Plus PM Array plates (Affymetrix, Santa Clara, CA) containing >54,000 probes, allowing for the analysis of >33,000 well-characterized human genes. Microarray analysis was conducted by the BIDMC Genomics, Proteomics, Bioinformatics and Systems Biology Center at the Beth Israel Deaconess Medical Center according to the standard Affymetrix protocol using the high-throughput Affymetrix GeneTitan system. The quality of hybridized chips was assessed using Affymetrix guidelines on the basis of perfect match (PM) probes mean, 3' to 5' ratios for beta-actin and GAPDH and values for spike-in control transcripts. Reproducibility of the samples was checked by using chip-to-chip correlation and signal-to-noise ratio (SNR) methods for replicate arrays using arrayQualityMetrics, a Bioconductor package in R [38]. All of the high quality arrays were included for unsupervised and supervised bioinformatics analysis. The transcriptome data along with information from this study have been deposited in the NIH Gene Expression Omnibus database at www.ncbi.nlm.nih.gov/geo under the accession number GSE66824.

### Data analysis

#### Self-Report Measures

Baseline differences between disease subgroups and between participants with and without follow-up data were compared by one-way ANOVA and Fisher’s exact test. Missing responses to individual questions from multi-question instruments were completed by single imputation from the observed covariance among all questions, adjusted for visit. Six of 87 IBDQ, 1 of 56 IBS-QOL, 1 of 56 IBS-SSI, 1 of 148 BPI-Severity, and 9 of 148 PCS instruments required imputation; in 12 of 18 cases for a single missing response. Self-report measures were analyzed in linear mixed models with fixed effects of visit (4 levels) and random participant-specific intercepts and slopes with unstructured covariance. Contrasts were used to estimate change from baseline to week-10 (primary endpoint) and to week-13 (short-term persistence). Changes in symptoms and markers of inflammation were compared by Pearson correlation. All participants were included in these analyses, including those lost to follow-up. Estimates from the mixed model would be unbiased if loss to follow-up were predictable from observed baseline data. As a sensitivity analysis, participants with missing data were assumed to have experienced no change from baseline. These 10-week change scores with imputed zeros were analyzed by one-sample Wilcoxon signed rank test.

#### Transcriptome Profile

Expression data were preprocessed using the Robust Multichip Average (RMA) method in R using Bioconductor and associated packages. RMA performed background adjustment, quantile normalization and final summarization of oligonucleotides per transcript using the median polish algorithm [37]. Batch effects were removed using ComBat, an empirical Bayes method [38]. Probes with absolute maximum expression intensity values of 10 were removed. When comparing pre- vs. post-intervention samples, differentially expressed genes were defined as those with p-value <0.05 by two-tailed Student’s t-test and absolute fold change greater than 1.2.

#### Gene Ontology (GO) analysis

To identify over-represented GO categories in differentially expressed genes, we used the Biological Processes and Molecular Functions Enrichment Analysis available from the Database for Annotation, Visualization and Integrated Discovery (DAVID) [39]. DAVID is an online implementation of the EASE software that produces a list of over-represented categories using jackknife iterative re-sampling of the two-tailed Fisher exact probabilities. A p-value gets assigned to each category on the basis of enrichments. Smaller p-values reflect increasing confidence in over-representation. The GO categories with p-values <0.01 and at least 3 genes were considered significant.

#### Pathway and Interactive Network analysis

Ingenuity Pathway Analysis (IPA 8.0, Qiagen) was used to identify the pathways and interaction networks significantly affected by genes that are altered after the RR-MBI in IBS and IBD patients. The knowledge base of this software consists of functions, pathways and network models derived by systematically exploring the peer reviewed scientific literature. A detailed description of IPA analysis is available at the Ingenuity Systems’ web site (http//www.ingenuity.com). It calculates a p-value for each pathway according to the fit of users’ data to the IPA database using one-tailed Fisher exact test. The pathways with p-values <0.01 were considered significantly affected.

For each network, IPA calculates a score derived from the p-value of one-tailed Fisher exact test [score = -log(p-value)] and indicates the likelihood of focus genes appearing together in the network due to random chance. A score of 2 or higher has at least a 99% probability of not being generated by random chance alone. The ability to rank the networks based on their relevance to the queried data sets allows for prioritization of networks with the strongest association with pre- to post-intervention changes.

#### Gene Set Enrichment Analysis

In addition to individual gene analysis, Gene Set Enrichment Analysis (GSEA) was performed. GSEA was used to determine whether *a priori* defined sets of genes showed statistically significant, concordant differences between pre- and post-intervention samples [40]. GSEA can be more powerful than single-gene methods for studying the effects of interventions such as the RR-MBI, in which many genes each make subtle contributions. The gene sets with nominal p-value (NPV) less than 0.005 and a False Discovery Rate (FDR) less than 7.5% after 500 random permutations were considered significantly altered. The enriched gene sets were merged into functional modules on the basis of overlap of significantly enriched genes using the Enrichment Map [41] Cytoscape Plugin: An Open Source Platform for Complex Network Analysis and Visualization [42].

#### Leading edge analysis

The significantly enriched genesets or pathways identified from the GSEA analysis described above can have significant overlap in terms of core-enriched genes that are potentially linked to enrichment of multiple genesets. “Leading edge” analysis considers genes shared across the gene sets which are most strongly associated with the phenotype or key effects of the intervention. We used leading edge analysis to cluster significant genesets and identify common genes which accounted for the core enrichment signal. The core set of genes identified by leading edge analysis is considered the most promising one to generate hypotheses about the mechanism of an intervention [40].

## Results

**Fig 1:**
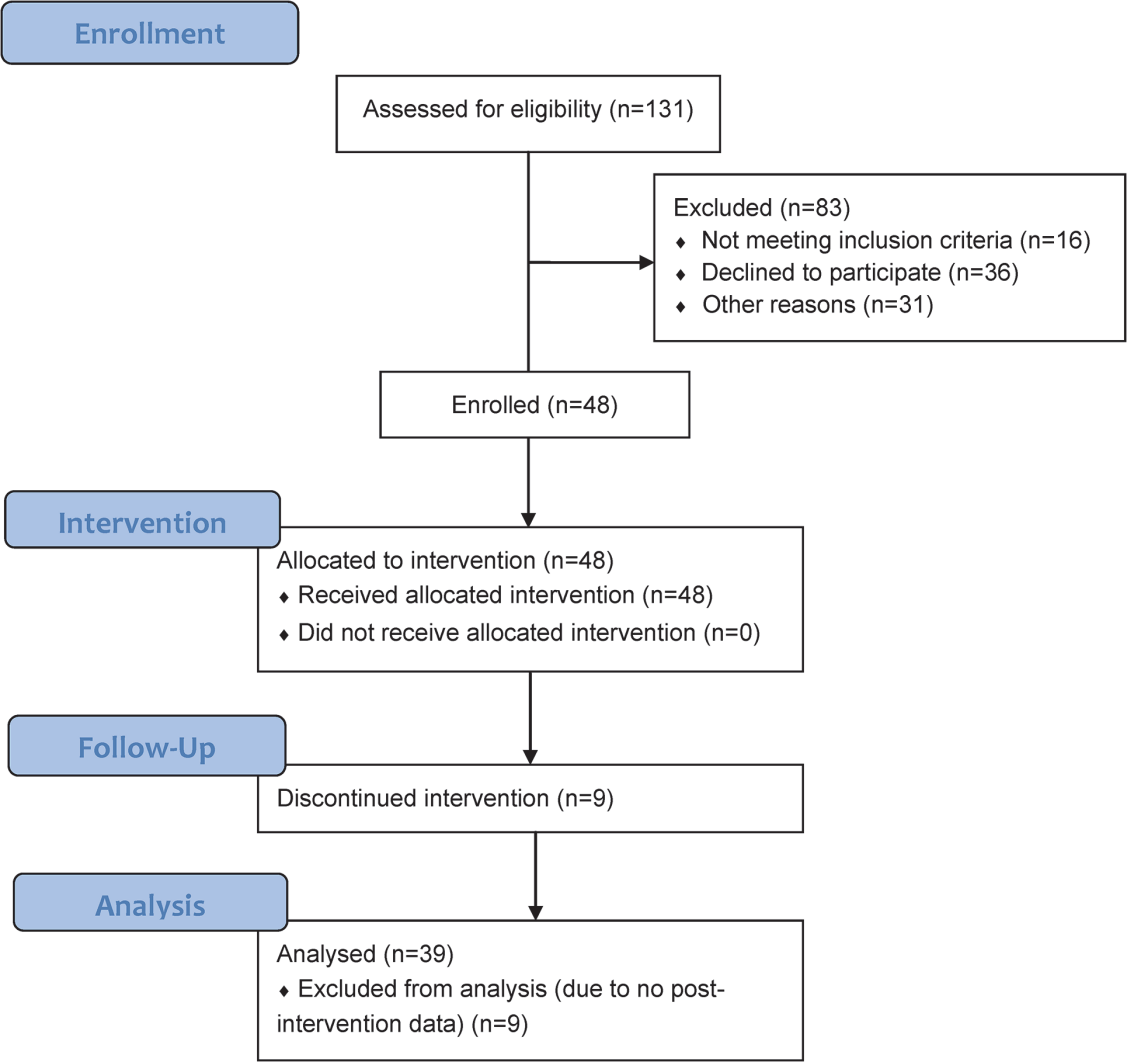
CONSORT Enrollment diagram.

Nineteen IBS (79% female, mean age 48.3±16.4) and 29 IBD (59% female, mean age 40.5±17.6) patients were enrolled. Four IBS and 5 IBD patients withdrew during the program after 1 to 3 sessions (Fig 1). Baseline characteristics of patients who withdrew and had no follow-up data had lower trait scores on the State Trait Anxiety Index and higher CRP levels but were not otherwise statistically different than those with all or some follow-up data. All participants were included in analyses. Additionally, there were no major differences in baseline characteristics between disease subtypes. The majority of patients were white (96%), single (54%), and had either some college or a bachelor’s degree (52%) or post-graduate education (38%) (Table 2). No adverse events due to the RR-MBI were reported.

**Table 2:** Baseline characteristics of participants.

		All Participants		Irritable Bowel Syndrome		Inflammatory Bowel Disease	
		N = 48		N = 19 (40%)		N = 29 (60%)	
		%	N	%	N	%	N
Gender (%)							
Female		67	32	79	15	59	17
Male		33	16	21	4	41	12
Race/Ethnicity (%)							
White		96	46	95	18	97	28
Other		4	2	5	1	3	1
Education (%)							
High-school		10	5	11	2	10	3
Some College/Bachelors		52	25	42	8	59	17
4-year degree		38	18	47	9	31	9
Marital status (%)							
Single/Never married		54	26	58	11	52	15
Married		38	18	32	6	41	12
Divorced/Widowed		8	4	10	2	7	2
Diagnosis Subtypes*							
IBS Subtypes	Constipation	15	7	37	7		
	Diarrhea	15	7	37	7		
	Mixed	10	5	26	5		
IBD Subtypes	Crohn's Disease	25	12			41	12
	Ulcerative Colitis	35	17			59	17

*No significant differences were found at baseline between subtypes of IBS and IBD, respectively.

### Self-report measures

**Fig 2:**
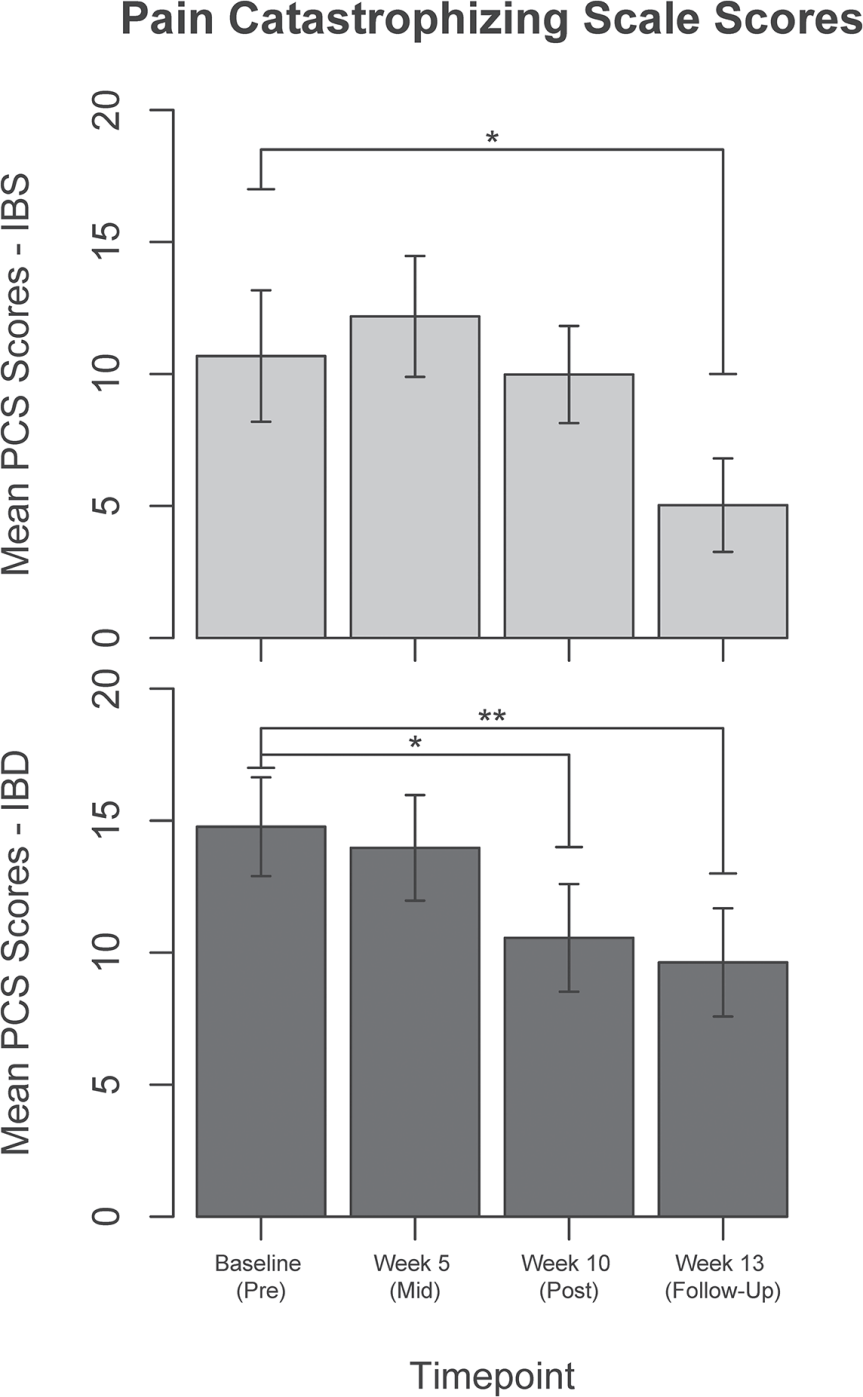
Pain Catastrophizing Scale scores at each of the four time points in Irritable Bowel Syndrome (IBS, light bars, top) and Inflammatory Bowel Disease (IBD, dark bars, bottom). *p<0.05, **p<0.01.

**Fig 3:**
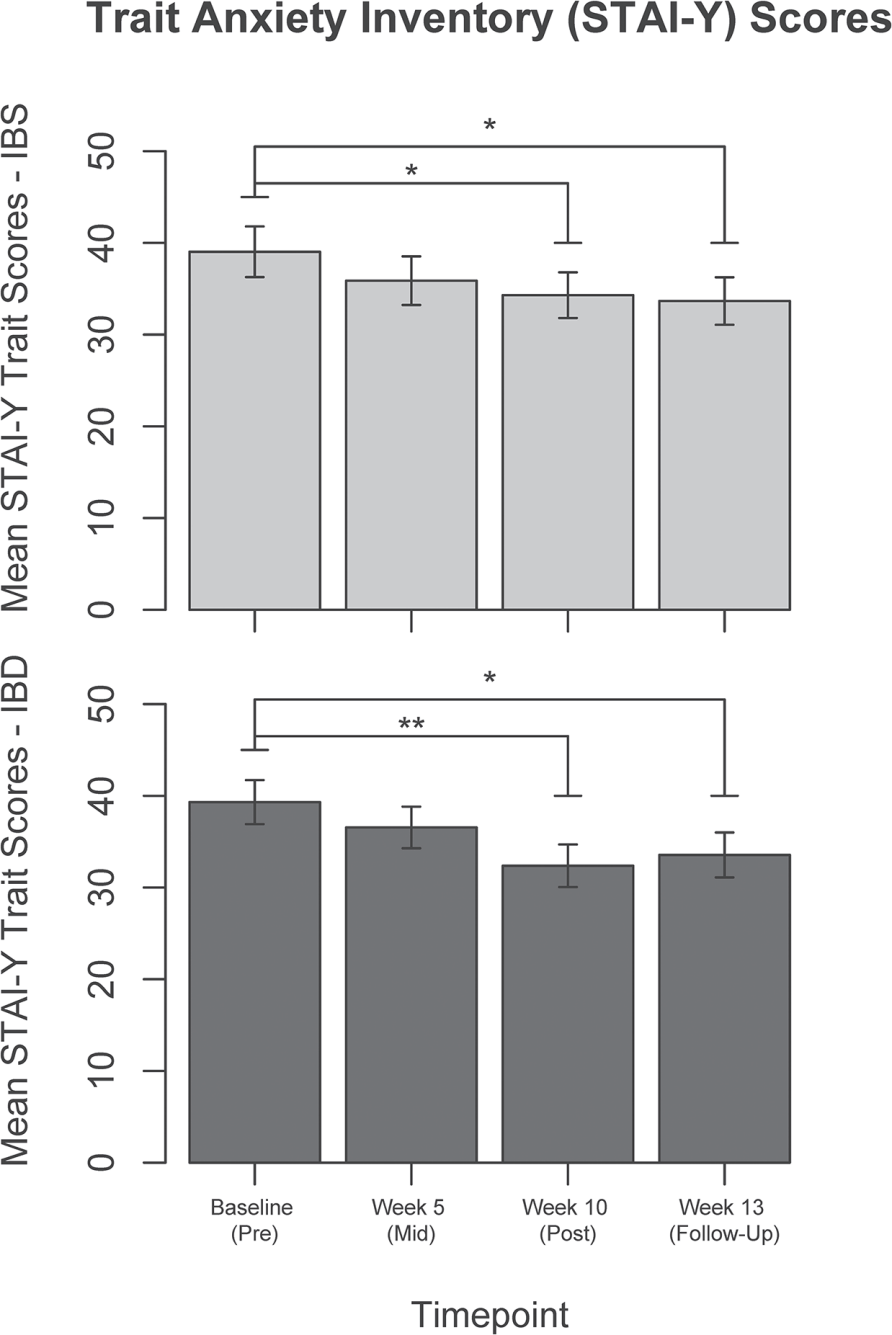
State-Trait Anxiety Inventory (STAI)—Trait scores at each of the four time points. STAI—Trait scores at each of the four time points in Irritable Bowel Syndrome (**IBS**, light bars, top) and Inflammatory Bowel Disease (**IBD**, dark bars, bottom). (*p<0.05, **p<0.01).

Mean changes in the self-report measures from pre- to mid- (week-5) and to post-intervention (week-10), as well as follow-up (week-13), are presented in Table 3 (for further details, including standard errors, please see S1 Table). PCS improved in both disease groups from baseline to week-13 (from 10.7 at baseline to 5.0 at week-13, p = 0.02 for IBS and from 14.8 to 9.6, p<0.01 for IBD, Fig 2). STAI trait anxiety improved in both groups from baseline to week-10 (from 39.0 to 33.7, p = 0.02 for IBS and from 39.3 to 33.6, p<0.01 for IBD). These improvements persisted at short-term follow-up (p = 0.02 for IBS and IBD, Fig 3). BPI measures did not change in either group (p = 0.42 for severity, p = 0.30 for interference).

**Table 3:** Outcome measures after intervention and at 3-week follow-up for irritable bowel syndrome (IBS) (N = 19) and inflammatory bowel disease (IBD) (N = 29).

**Irritable Bowel Syndrome**	Baseline (Pre-Intervention)	Week 5 (Mid-Intervention)	Week 10 (Post-Intervention)	Week 13 (Short-Term Follow-Up)	Overall P-value
Pain Catastrophizing Scale (PCS)	10.7	12.2	10.0	5.0*	0.021
State-Trait Anxiety Inventory(STAI-Y)—State Anxiety	37.2	35.8	31.6	30.8	0.18
State-Trait Anxiety Inventory(STAI-Y)—Trait Anxiety	39.0	35.9	34.3*	33.7*	0.034
IBS Quality of Life (IBS-QOL)	67.1	71.5	74.8*	80.6***	0.009
IBS Symptom Severity Index (IBS-SSI)	215.0	153.7**	127.5***	147.1*	0.002
**Inflammatory Bowel Disease**					
Pain Catastrophizing Scale (PCS)	14.8	14.0	10.6*	9.6*	0.003
State-Trait Anxiety Inventory(STAI-Y)—State Anxiety	36.9	36.0	31.6	31.7	0.14
State-Trait Anxiety Inventory(STAI-Y)—Trait Anxiety	39.3	36.6	32.4**	33.6*	0.012
IBD Questionnaire (IBD-Q)	171.2	172.0	185.0**	184.3*	0.01

*P<0.05

**P<0.01

***P<0.001

Indicate mixed model analysis estimating change from baseline scores

**Fig 4:**
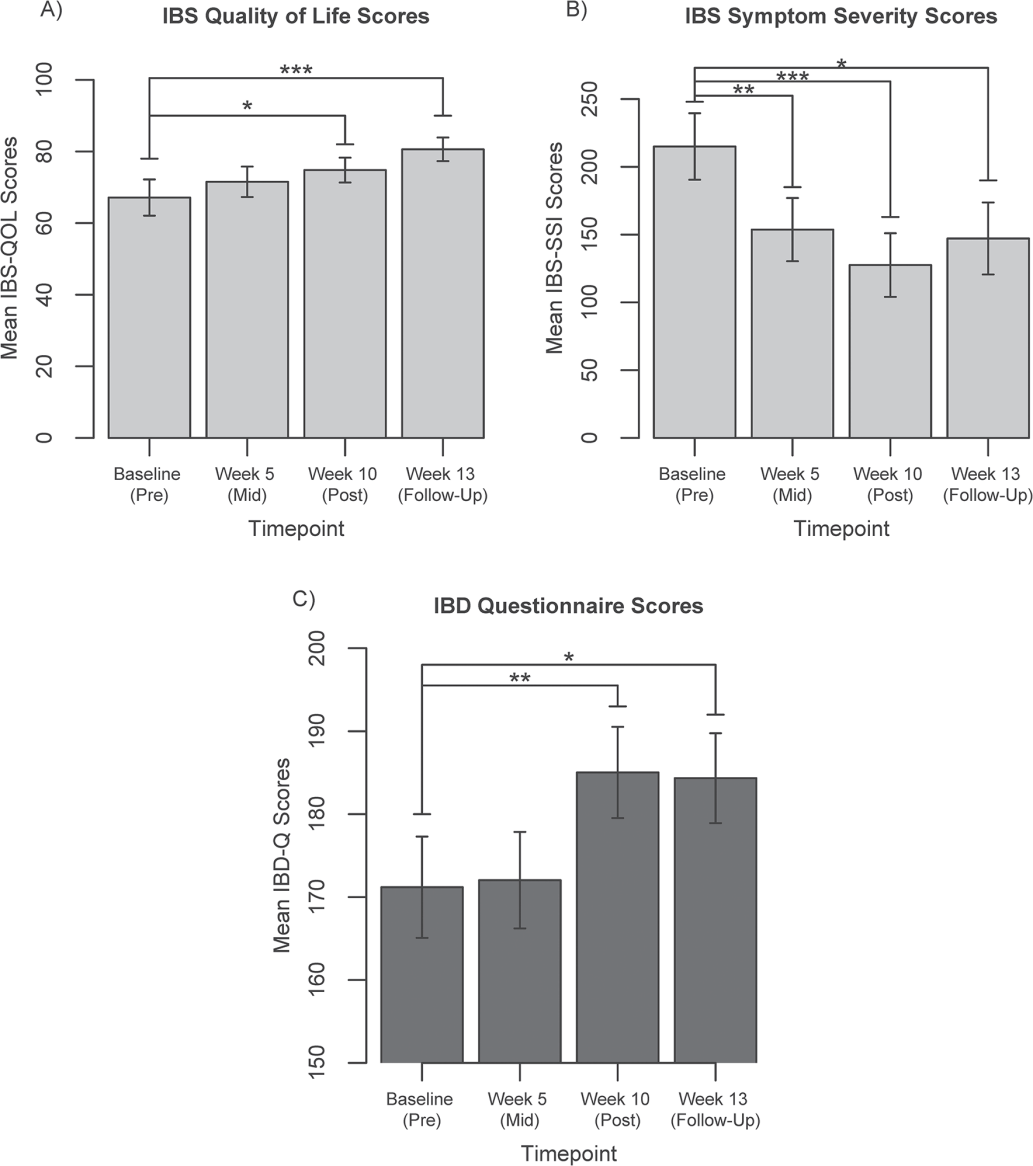
Disease-specific quality of life and symptom measures at each of the four time points. A) Irritable Bowel Syndrome Quality of Life (**IBS**-QOL) scores; lower scores indicate improvement. B) Irritable Bowel Syndrome Symptom Severity Index (**IBS**-SSI) scores; lower scores indicate improvement. C) Inflammatory Bowel Disease Questionnaire (**IBD**-Q) scores; higher scores indicate improvement. *p<0.05, **p<0.01, ***p<0.001.

IBS-QOL scores increased from a mean of 67.1 at baseline to 74.8 at week-10 (p = 0.01), and to 80.6 at week-13 (p<0.001) suggesting clinical improvement (Fig 4A). IBS-SSI scores decreased significantly, demonstrating improvement in symptoms, from a mean of 215 at baseline to 154 at week-5 (p<0.01), 128 at week-10 (p<0.001), and 147 at week-13 (p = 0.01, Fig 4B). IBD-Q scores increased, suggesting clinical improvement, from a mean of 171 at baseline to 185 at week-10 (p<0.01) and remained at 184 at week-13 (p = 0.02, Fig 4C).

In both disease groups, estimated changes over 10 weeks were still significant when treating all drop-outs or missing assessments as unchanged from baseline.

### Biological Measures

#### Serum inflammatory markers

ESR and CRP did not change significantly for either IBS or IBD patients. In addition, no changes in psychological measures correlated with ESR/CRP changes.

#### Baseline gene expression differences between IBS and IBD

**Fig 5:**
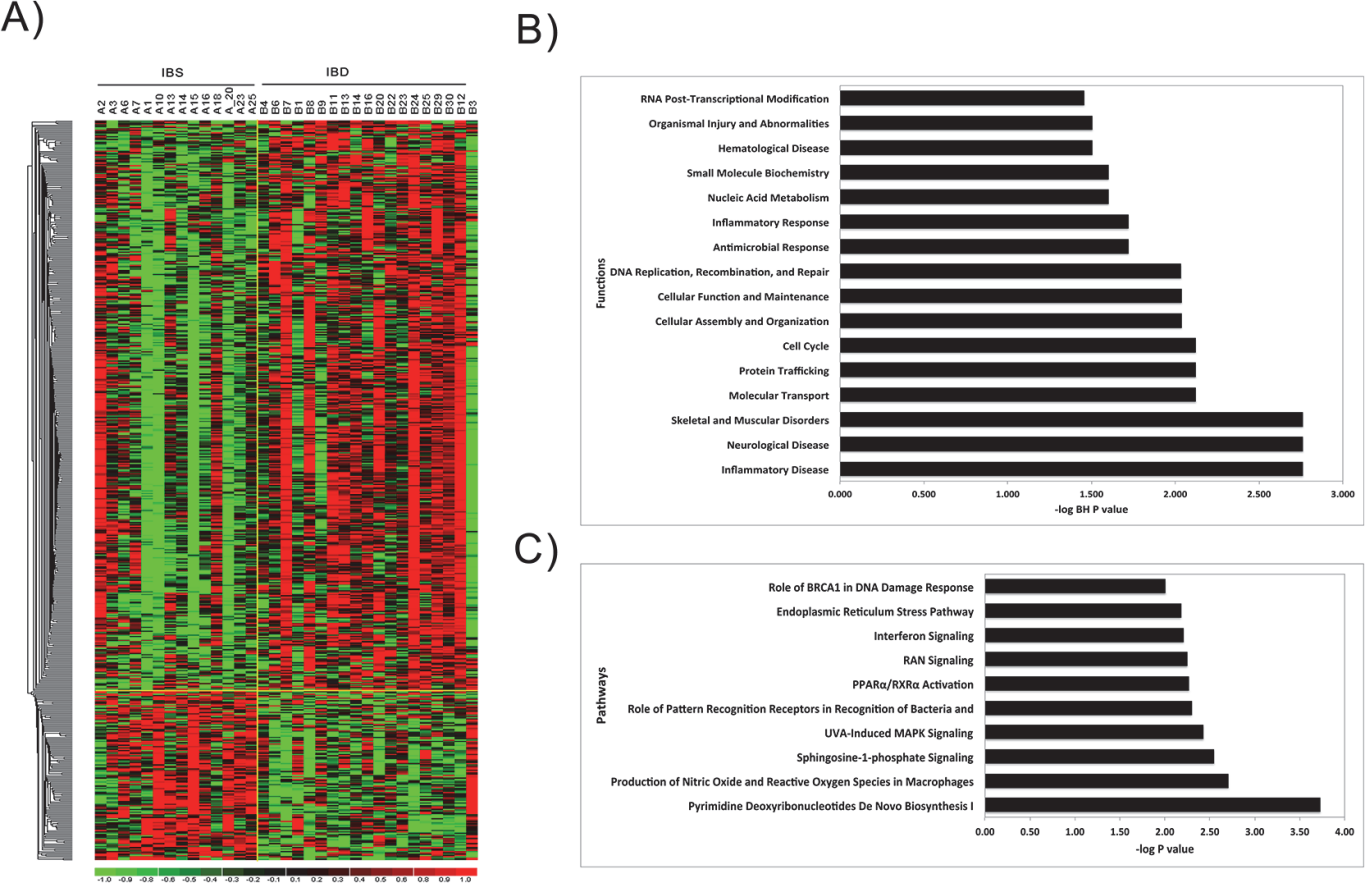
Transcriptional differences between IBS and IBD patients at baseline, pre-intervention, shown by A) a heatmap of significantly differentially expressed genes, B) functional categories enrichment analysis, and C) pathways enrichment analysis. A) Relative Gene expression difference between IBD and IBS for significantly differentially expressed genes are shown with a pseudocolor scale (-1 to 1) with red color denoting higher gene expression among IBD patients and green color denoting higher expression among IBS patients. The rows represent the genes and columns represent individual patients. A large fraction of differentially expressed genes were found to be upregulated in **IBD** patients at baseline relative to **IBS** patients. B) Selected top functional categories of differentially expressed genes. The Y–axis represent functional categories and X-axis-log transformed P value (i.e., a value of 2 represents a P value of. 01), C) Selected pathways of by differentially expressed genes. The Y—axis represent pathways and X-axis-log transformed P value.

At baseline, 564 significantly differentially expressed transcripts (p≤0.05 and absolute fold change [AFC] ≥1.2) distinguished IBS from IBD patients (S2 Table). A heatmap depicting the pattern of these differentially expressed transcripts is shown in Fig 5A and suggests that in IBD significantly more of these differentially expressed genes are upregulated in comparison to IBS. Functional enrichment analysis of these differentially expressed genes indicates significant overrepresentation (multiple test corrected [43] p<0.05) in inflammatory and antimicrobial response, as well as inflammatory and hematological disease, RNA post-transcriptional modification, cell cycle, cell proliferation, and molecular transport (Fig 5B). Pathway analysis suggests that some of these genes are linked to DNA damage response, MAPK signaling, PPAR-γ activation, interferon signaling and endoplasmic reticulum stress pathways (Fig 5C).

#### Genomic alterations induced by the RR-MBI in IBS patients

**Fig 6:**
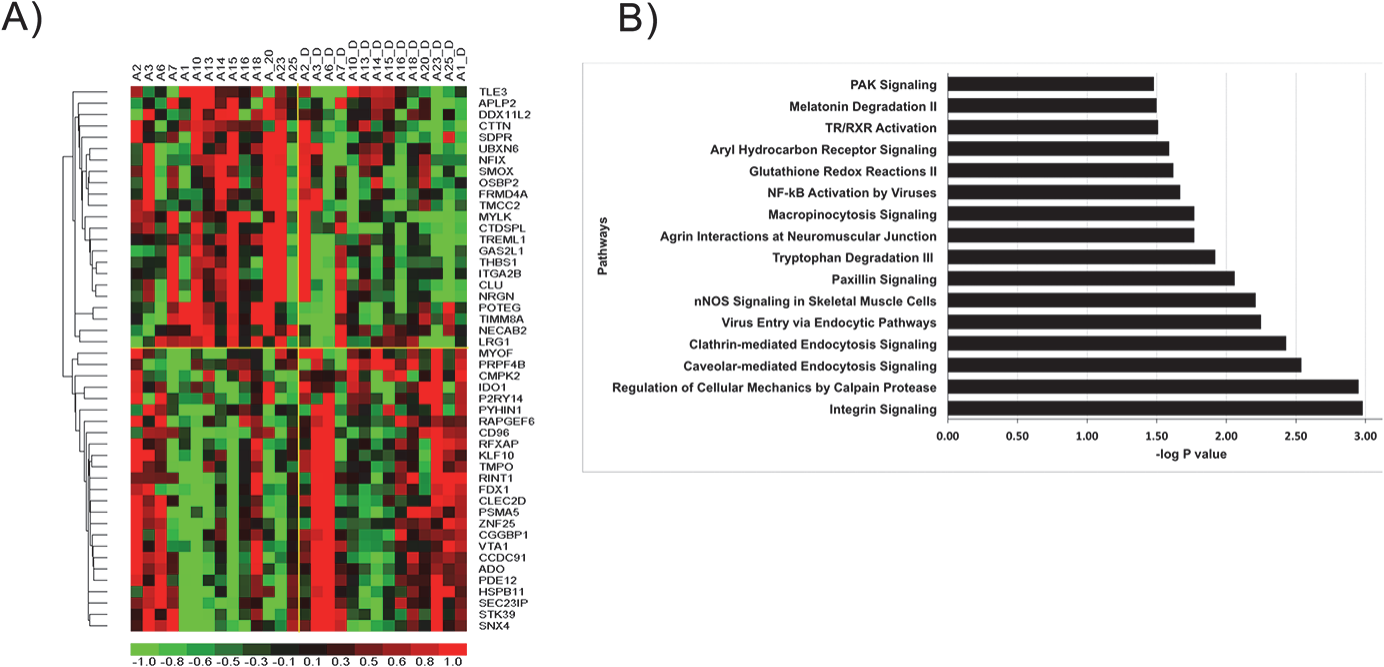
Transcriptional changes and significantly affected pathways for IBS patients pre- to post-mind body intervention. A) Heatmap of selected top differentially expressed genes identified by comparing pre- to post-mind body intervention transcriptional profiles. Gene expression is shown with a pseudocolor scale (-1 to 1) with red color denoting increased gene expression post-intervention and green color denoting decreased expression post-intervention. The rows represent the genes and columns represent individual subjects in **IBS** group. B) Top pathways of genes with altered expression among **IBS** and IBD patients. Pathway enrichment analysis was performed on differentially expressed genes and significance of effect on pathway was determined using Fisher’s Exact Test p value. Each bar represents a significantly enriched pathway. The p value is depicted as —log10 (p value) on primary X-axis.

A total of 191 transcripts were significantly differentially expressed (AFC≥1.2 and p≤0.05) post-intervention compared to pre-intervention. A heatmap depicts the relative expression levels of the selected top differentially expressed genes (Fig 6A). The highest-ranking GO clusters of biological processes and metabolic functions enriched in these RR-MBI response genes based on analysis by DAVID are shown in S4 Table. Stress/inflammatory response is the most highly enriched GO cluster and includes the selected processes of cellular response to stress and inflammatory response. Pathway enrichment analysis using IPA tools identified significant associations (Fisher’s Exact test p-value <0.05) with integrin and endocytosis signaling, nNOS signaling, paxillin signaling, tryptophan degradation signaling, NF-κB activation and glutathione redox reaction II (Fig 6B).

#### Interactive Network analysis of RR-MBI-induced gene expression changes in IBS identifies networks and key nodes related to cell cycle, cellular proliferation, hematological systems, and inflammation (Details S1 Protocol)

The analysis of interactive network based on number of interactions identified multiple key nodes that include UBC, PI3 Complex, TNF, AKT, NF-κB complex, TP53 and IFNβ.

#### The RR-MBI upregulates gene sets related to cell cycle, electron transport chain and DNA Damage and Repair in IBS: Gene set enrichment analysis comparing expression changes from week-10 to baseline

GSEA analysis identified 78 pathways that were significantly (p<0.005, FDR<0.075) altered following the RR-MBI (S1 Fig). The significantly altered pathways include a cluster related to cell cycle (e.g., mitotic cell cycle, cell cycle checkpoint, cell cycle M-G1 transition phase), DNA Damage and Repair (e.g., nucleotide excision repair, telomere extension, spliceosome), primary metabolism (e.g., amino acid metabolism, cholesterol biosynthesis), electron transport chain and muscle contraction. The GSEA analysis shows that cell cycle and DNA damage-related pathways are mostly upregulated in IBS patients after 10-weeks of RR-MBI (S2 Fig).

#### Genomic alterations induced by the RR-MBI in IBD patients

**Fig 7:**
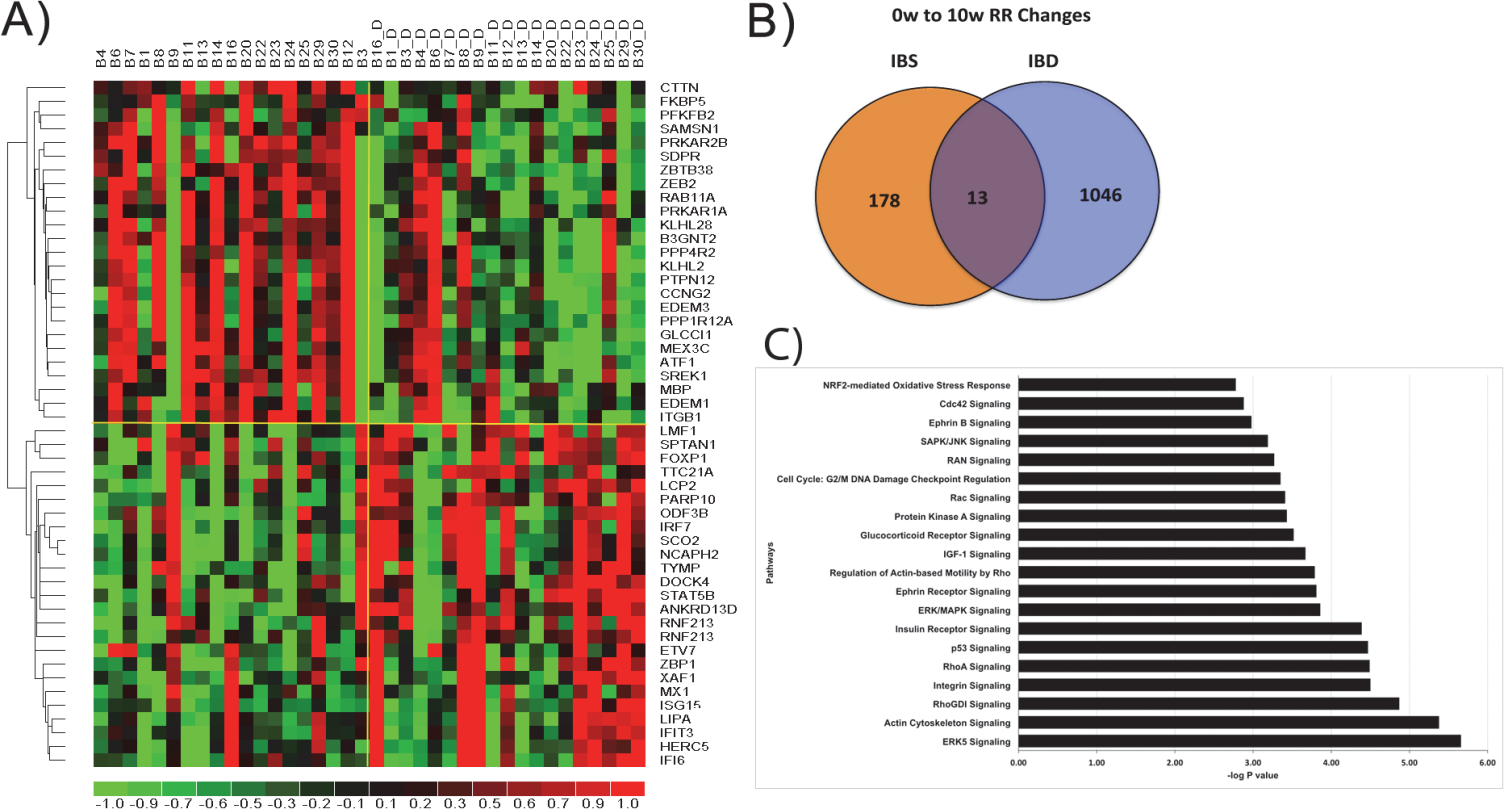
Transcriptional changes and pathways modulated pre- to post-RR-MBI among IBD patients. A) Heatmap of selected differently expressed genes identified by comparing pre- to post-mind body intervention transcriptional profiles of **IBD** patients. Gene expression is shown with a pseudocolor scale (-1 to 1) with red color denoting increased fold change in gene expression and green color denoting decrease. The rows represent the genes and columns represent individual subjects in **IBD** group. B) Venn Diagram depicting common genes between **IBS** and **IBD**. Only 13 genes were commonly altered between IBS and IBD. **C)** Top pathways significantly affected by differentially expressed genes in **IBD** group. The statistical significance of effect on pathways was calculated using Fisher’s Exact Test. The pathways with P value <. 05 were considered significantly effected. Each bar represents a significantly enriched pathway. The p value is depicted as —log10 (p value) on the x-axis.

The RR-MBI resulted in significant differential expression of 1,059 transcripts in IBD patients. Around 73% of these genes were downregulated. The number of transcripts with altered expression following the RR-MBI in IBD was much higher (1059) than in IBS (191). A heatmap of selected genes altered by the RR-MBI is shown in Fig 7A, revealing a clear distinction between the subjects before and after the RR-MBI. Among the genes that were reduced in expression upon completion of the RR-MBI, there are several genes which have been directly implicated in inflammatory processes that play a critical role in IBD, particularly several genes involved in interferon regulation and signaling. For example, cortactin (CTTN, Fig 7A), an actin-binding protein, is required for endothelial barrier functions, vascular permeability, neutrophil recruitment, and leukocyte adhesion and extravasation during inflammation [44]. The GTPase RAB11A (Fig 7A) is involved in recruitment of TLR4 to phagosomes and IRF3 signaling, both of which are key events in the innate immune response and host defenses against bacterial infection and are highly relevant for IBD [45]. Several endoplasmic reticulum (ER) stress markers involved in the unfolding protein response, such as EDEM1 and EDEM3 (Fig 7A), are reduced upon completing the RR-MBI. This suggests that the RR-MBI reduces ER-associated degradation (ERAD) activity. Interestingly, upregulation of markers of ERAD activity such as EDEM1 has been observed in actively inflamed IBD [46].

Among the genes that are upregulated in response to the RR-MBI in IBD patients is a key transcriptional regulator for generating quiescent naïve T cells, FOXP1 [47] (Fig 7A). Reduced FOXP1 expression leads to T cell proliferation and activation which is directly involved in immune response and inflammation linked to IBD. Thus, it appears that RR-MBI-mediated upregulation of FOXP1 may reduce T cell activation and potentially attenuate inflammation. Similarly, enhanced expression of IRF7 (Fig 7A), a transcriptional regulator of interferon expression, in response to the RR-MBI may limit the inflammatory sequelae of IBD [48]. Another upregulated gene of interest is STAT5B (Fig 7A). STAT5B expression is reduced in the colon of Crohn’s disease patients and STAT5B deficient mice are more susceptible to colitis [49]. STAT5B has been postulated to reduce inflammation and to play a protective role in inflammation. STAT5B activation increases the suppressor function of regulatory T cells (Tregs) while inhibiting effector T cells (Teffs); furthermore, a reduction in Tregs has been observed in IBD [50]. Thus, the increase in STAT5B expression in response to the RR-MBI in IBD patients may reduce inflammatory processes and enhance Treg function in IBD. MX1 (Fig 7A) is also upregulated by the RR-MBI in IBD patients. MX1 downregulation has been shown to lead to increased cytokine and chemokine production concomitant with recruitment of monocytes and granulocytes and an increase in inflammation [51].

Comparing the differentially-expressed genes in the two patient groups demonstrated that 13 transcripts were affected by the RR-MBI in both IBS and IBD patients, as shown in the Venn diagram in Fig 7B. The list of 13 genes affected in common (S3 Table) includes genes already linked with IBD (e.g., FKBP5, ITGB3).

Gene ontology (GO) enrichment analysis with DAVID identified ATP nucleotide binding, cellular processes, cell death/apoptosis, immune system processes, immune cell activation, regulation of immune system, kinase activity, glucose metabolism, and stress response as GO categories with highly significant overrepresentation of differentially expressed genes (Table 4). Most interestingly in the context of IBD and the RR-MBI, leukocyte, T cell and B cell activation, as well as NF-κB regulation, immune and defense responses and cellular response to stress were significantly affected biological processes. Unlike the IBS group, genes with significant changes in expression in the IBD group were not linked to cell cycle and cellular movement related processes.

**Table 4:** Gene-Ontology (GO) enrichment analysis of genes altered in IBD patients after the RR-MBI.

Major GO Cluster	Enrichment Score	Selected Processes	P value
**IBD**			
**ATP and Nucleotide Binding**	5.99		
		Nucleotide binding	1.45E-08
		ATP binding	5.52E-07
		Protein serine/threonine kinase activity	2.44E-06
**Protein transport**	5.46		
**Cellular process**	4.42		
		Primary metabolic process	2.39E-07
		Nucleobase, nucleoside, nucleotide and nucleic acid metabolic process	4.73E-04
**Negative regulation of cellular process**	4.25	Negative regulation of transcription	2.45e-05
** Cell death/apoptosis**	3.66		
		Regulation of apoptosis	7.93E-06
		Regulation of programmed cell death	1.07E-05
		Induction of apoptosis	0.037
		Induction of programmed cell death	0.038
**Macromolecule catabolic process**	2.88		
		Cellular protein catabolic process	1.31E-04
		Ubiquitin-dependent protein catabolic process	2.84E-04
		Protein ubiquitination	0.005
**Regulation of kinase activity**	2.71		
		Regulation of protein kinase activity	0.003
		Regulation of MAP kinase activity	0.019
**Nucleoside-triphosphatase activity**	2.55		
		ATPase activity	0.050
**Immune system process**	2.42		
		Regulation of immune system process	5.25E-05
		Regulation of myeloid cell differentiation	1.19E-04
**Immune cell activation**	2.39		
		Leukocyte activation	4.44E-05
		T cell activation	0.019
		B cell activation	0.030
**Vesicle-mediated transport**	2.24		
		Vesicle-mediated transport	8.12E-05
**Regulation of binding**	2.2		
		Positive regulation of binding	0.001
		Positive regulation of NF-κB transcription factor activity	0.010
**Regulation of immune system process**	2		
		Positive regulation of immune response	0.010
		Positive regulation of defense response	0.024
**Glucose metabolism**	2		
		Energy reserve metabolic process	0.004
		Glucose metabolic process	0.016
**Stress response**	1.97		
		Cellular response to stress	0.002
		Response to stress	0.010

The enrichment score for each biological group indicates its importance (enrichment) and was calculated by taking the geometric mean of enrichment p-values (EASE scores) for each GO term associated with the gene members in the group. An enrichment score of 2 is equivalent to a non-log p-value of 0.01.

IPA analysis of the differentially expressed genes in IBD following the RR-MBI revealed significant enrichment for cellular growth and proliferation (e.g., ERK5 Signaling, ERK/MAPK, RhoA, Integrin, P53, Cdc42, and Actin-cytoskeleton signaling), inflammation and stress response (e.g., IGF1 and glucocorticoid receptor signaling, and NRF2-mediated oxidative stress response), as well as insulin-signaling related pathways (Fig 7C).

#### Interactive network analysis of RR-MBI-induced gene expression changes in IBD identifies networks and key nodes related to cell morphology, inflammation and apoptosis

**Fig 8:**
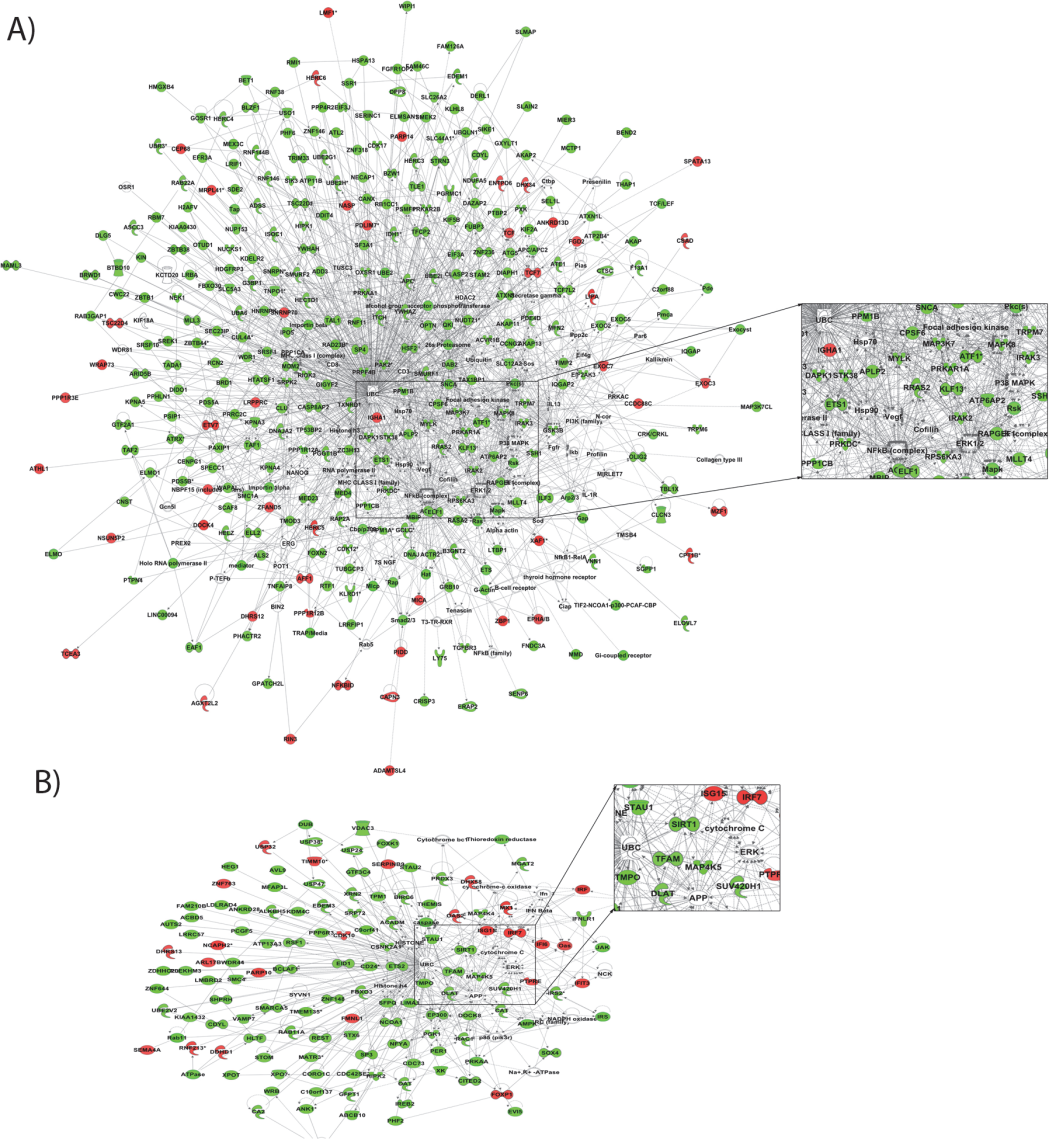
Network representation of the biological functions significantly altered by 8-weeks of RR-MBI in IBD patients. Networks shown: A) Cellular morphology and tissue development related genes with UBC, MAPK8, NF-κB and ERK1/2 as primary regulatory nodes; B) Genes involved in cell death, apoptosis and inflammation with UBC, APP and IRF7 as a critical regulatory node. We used the Ingenuity Pathways Analysis tool (IPA 8.0) to generate the networks of genes altered by RR-MBI in **IBD** patients and merged the major networks with obvious related functions. Each node represents a gene and each edge represent a molecular interaction. The intensity of the node color indicates the degree of upregulation (red) and downregulation (green), while white nodes indicate non-modified genes that may be affected in a non-transcriptional manner.

Interactive network analysis with the Ingenuity Knowledge Base (Qiagen) identified >20 significantly (i.e., score >20) altered interactive networks linked to cell morphology and tissue development, apoptosis and inflammation. Networks with related functions were merged to generate an integrated picture of biological relationship for better understanding of the underlying mechanism of changes associated with an RR-MBI. We merged the networks “cell and tissue morphology,” “apoptosis,” “hematological,” and “inflammation” to generate a global overview of the effect of the RR-MBI on IBD (Fig 8). The genes altered by the RR-MBI in IBD were mostly (~85%) downregulated, suggesting that the RR-MBI serves to downregulate or inhibit apoptosis and inflammation related processes. On the basis of network analysis, key focus hubs included: UBC, NF-κB, ERK1/2, MAPK8, MAPK, p38 MAPK, MAP3K7 and APP.

#### RR-MBI downregulates gene sets related to cell cycle and the immune system in IBD

**Fig 9:**
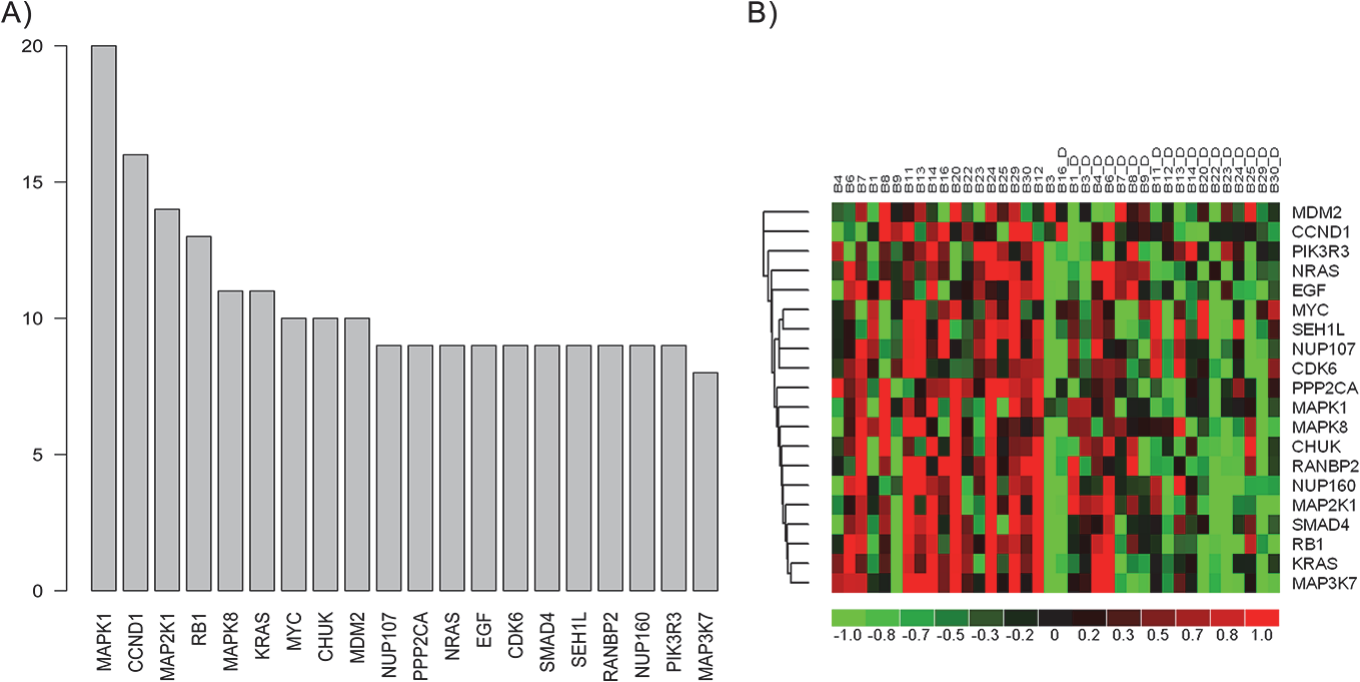
Identification of potential key genes responsible for delivering the beneficial effects of RR-MBI in IBD identified using Gene Set Enrichment Analysis and leading edge analysis. A) Bar graph depicting the abundance of genes in significantly enriched geneset (S2 Fig). B) Heatmap of most abundant genes in **IBD** patients depicting the pattern of downregulation after the RR-MBI.

GSEA analysis identified 60 pathways that were significantly (p<0.005, FDR<0.075) altered following the RR-MBI (S3 Fig). The significantly altered pathways include a cluster related to cell cycle (e.g., mitotic cell cycle, G1 phase, G2 phase, G1-G2-M phase, P53 signaling), apoptosis (e.g., apoptosis, BAD pathway), immune system (e.g., TLR, IFN, TNFR1, PPARA, P38MAPK, IL1R pathways), extracellular matrix related pathways (e.g., TGFB and BMP), insulin, and hypoxia. Further to identify the core subset of genes from significantly enriched genesets that is strongly associated with effect of the RR-MBI on IBD, we performed leading edge analysis. As noted above, leading edge analysis identifies a core subset of genes that accounts for the enrichment signal, which can lead to hypotheses about the biological mechanism of the intervention. Leading edge analysis identified multiple cell cycle and apoptosis-related molecules (MAPK1, CCND1, MAP2K1, MAPK8) as associated with the RR-MBI (Fig 9A). All of these molecules were significantly downregulated after the RR-MBI (Fig 9B). Some of these molecules were also identified as key focus molecules related to cell cycle and apoptosis (MAPK, MAPK8, MAP3K7) in the interactive network analysis.

## Discussion

While genetic predisposition, microbiome, and transcriptional profiling studies in IBS and IBD have indicated a role of oxidative stress related pathways [52–56], little progress has been made exploring the beneficial effects of mind-body interventions (MBIs) in these diseases. Our group has previously shown that regular elicitation of the relaxation response (RR) alters the expression of inflammatory molecules [27,28]. Building on these findings, we performed what we believe to be the first study to determine psychological, inflammatory, and genomic markers of a mind-body intervention in a combined group of patients with IBS and IBD.

In this open, uncontrolled study of an RR-MBI for GI disorders, mixed groups of patients with IBS and IBD jointly participated in a 9-week group intervention. In both IBS and IBD patients, trait anxiety decreased significantly over the course of the intervention; this improvement was maintained during the follow-up period. Pain catastrophizing also decreased significantly in both IBS and IBD patients but, interestingly, the timing was different: Whereas IBD patients had significantly decreased pain catastrophizing at the end of the intervention which was maintained during the follow-up period, IBS patients did not have significantly decreased pain catastrophizing scores until the completion of the follow-up period. There were also improvements in disease-specific measures of both symptom severity (for IBS) and quality of life (for both IBS and IBD). These findings suggest that an RR-MBI can be effective in decreasing distress and improving quality of life in both IBS and IBD.

The combined clinical group exposure to an MBI was a unique way to streamline resources for seemingly disparate clinical populations. Because the financial resources and access to personnel needed to deliver behavioral interventions are often lacking in GI clinics, it is common for these treatments to be unavailable. The group setting allows for more resource-efficient delivery of care to a broader audience of patients with gastrointestinal disorders but, despite the evidence presented here for its value, it continues to be unclear whether it provides the same benefits as individual treatment.

In addition to improvements on self-report measures, our data provide the first evidence that an RR-MBI in IBS and IBD patients is associated with specific gene expression changes. Although many of the RR-specific gene expression changes differed significantly between IBS and IBD patients and significantly more gene expression changes were observed in IBD than in IBS, changes in upstream and downstream targets of NF-κB (e.g. MAPK, P38 MAPK, MAPK8) after the RR-MBI were common to both IBS and IBD, supporting the notion that RR elicitation may counteract the effects of stress on IBS and IBD through common changes in stress-related gene expression. There is a growing understanding of central (i.e., brain) changes brought about by behavioral interventions in GI disorders. For example, Lowen et al. recently demonstrated that the abnormal processing and enhanced perception of visceral stimuli in IBS can be normalized by both hypnotherapy and an educational intervention [57]. It is also known that placebo can produce adequate relief of IBS symptoms and this is a limitation to our study design with respect to IBS. In one study serum levels of TNF-related weak inducer of apoptosis (TWEAK) at baseline were higher (p = 0.0144) in IBS patients who reported adequate relief and in particular in those who received the placebo treatment [58]. This suggests that modulation of inflammation and apoptosis may be common to brain-based improvements in IBS, including those related to placebo. The transcriptome changes we demonstrate here suggest a mechanism by which MBIs can bring about concomitant peripheral changes.

Despite clear changes in both quality of life and gene expression with the RR-MBI, there were no clear changes in ESR and CRP, commonly used markers of gross inflammation. ESR and CRP are relatively blunt markers of active inflammation and frequently respond rather slowly to changes in inflammatory processes. For the IBS patients, who suffer from a disorder that does not involve gross inflammation, it may not be surprising that these markers showed no changes. For the IBD patients, who suffer from a disorder that does involve gross inflammation, the key may have been their inflammatory state before and during the study. They were clinically stable—in remission from severe inflammation despite the fact that they were still suffering from symptoms related to IBD. (Indeed, clinically there can often be a disconnect between gross inflammation and symptoms in IBD.) More specific markers, such as fecal calprotectin or lactoferrin, responsive to more subtle inflammatory changes, may be able to reveal underlying changes, whether for IBD in remission or for hypothesized micro-inflammation in IBS. In future studies, use of placebo control conditions and specific inflammatory markers will be used. The choice of such markers could be informed both by advances in the understanding of inflammation in IBS and IBD and by the inflammation-related genomics findings we report here.

The comparison of baseline gene expression profiles of IBS and IBD groups indicated significant differences in the expression profile of genes linked to inflammation, suggesting that the IBD and IBS populations differ in baseline levels of inflammatory component compared. Associated with an RR-MBI and concomitant daily RR elicitation, transcriptome changes were identified in both the IBS and IBD groups. Some of these transcriptome changes, including those in inflammation and immune pathways, overlap with transcriptome changes induced by RR elicitation in a healthy population [27,28]. This suggests that the RR modulates a core set of common inflammation and immune pathways in both healthy and disease populations as one mechanism by which it has its beneficial effects specifically in IBD population.

In IBD patients, the RR-MBI reduced expression of various genes that are directly involved in key pathophysiological processes and pathways linked to IBD development and progression. This included a whole set of genes linked to interferon regulation and signaling, genes associated with inflammation, endothelial barrier functions, vascular permeability, neutrophil recruitment, and leukocyte adhesion and extravasation, innate immune response genes, T cell differentiation genes as well as several ER stress markers. Most importantly, the direction of the expression changes of these genes impacted by the RR-MBI appears to counter the inflammation and immune system disturbances observed in IBD and is expected to dampen the deregulated immune responses.

In the IBS group, participation with the RR-MBI was significantly associated with changes in a network enriched in genes linked to the cell cycle, cellular proliferation and inflammation; the NF-κB complex, MAPK and TNF were key focus molecules suggesting their key role in relaying effects of RR in the IBS population.

Similar Interactive network analysis in the IBD group network focus hubs are formed by inflammation-related genes (e.g., NF-κB) and kinases (e.g. ERK1/2, MAPK8, MAPK, MAP3K7). The genes forming focus hubs are considered the most critical for the overall function of the network. This suggests that NF-κB activity, along with altered expression of its up- and down-stream regulators, was central to the changes associated with the RR-MBI. Multiple studies have shown that enhanced NF-κB activity leads to increased expression of multiple cytokines that play a central role in the onset and progression of IBD [59]. The interactive network analysis shows that most of target genes for key focus molecules are downregulated by RR elicitation in IBD population, suggesting a parallel decrease in inflammation.

To further refine and strengthen our finding, we performed a complementary analysis using GSEA on the transcriptome changes in IBS and IBD subjects, which resulted from participation in an RR-MBI. In IBS patients GSEA analysis identified significant upregulation of multiple cell cycle related DNA Repair and mRNA processing pathways (S2 Fig). On the other hand, in IBD patients GSEA analysis identified significant downregulation of multiple cell cycle-, immune system- and apoptosis-related pathways, corroborating the results from the traditional individual gene based analysis. Further analysis of genes from these pathways identified multiple NF-κB upstream kinases (MAPK1, MAP2K1, MAPK8) as molecules that may be associated with effects of an RR-MBI. Studies using both gene expression profiling and protein-based assays have shown activation of Mitogen-activated protein kinases (MAPK) to be important in IBD [59]. AN RR-MBI might be relaying its beneficial effects by downregulating expression or deactivating MAPK in such a way that multiple pro-inflammatory cytokines are downregulated.

This single-center, uncontrolled, open-label study of a multimodal intervention has inherent limitations. First among these is a limitation common to any multimodal intervention such as the RR-MBI used here, namely, the inability to pinpoint any particular component of the intervention (e.g., RR-elicitation practice, building cognitive skills, or interaction with a group of fellow sufferers) as the “active ingredient.” Future work will be needed to determine whether certain components of the intervention are more important than others or whether the components combine to provide synergistic benefit. Furthermore, without the benefit of a randomized trial, it is impossible to say more than that the changes reported here are associated with participation in the RR-MBI. Still, the findings here do provide a first look at the effects MBI in general may have on psychological, inflammatory and genomic markers in IBS and IBD patients. Although our gene expression data was drawn from peripheral blood and not from either the brain or gut (where one would expect the interface between MBI and GI disorders), a growing body of evidence suggests the ability of mind-body and other behavioral interventions [27–30] to affect gene expression in peripheral blood mononuclear cells. Furthermore, there is evidence that peripheral blood gene expression is moderately correlated with gene expression levels in other tissues, including brain [60,61]. This correlation may permit identification of promising PBMC biomarkers for stress-related neuroimmunological disorders and their mind body management [62]. These results provide a platform for starting clinical trials to validate the role of focus genes and pathways in delivering the beneficial effects of RR-MBI in IBS and IBD.

## Conclusion

In this uncontrolled pilot study, improvement in symptoms and quality of life in IBS and IBD patients was associated with receiving a group RR-MBI. With scarce health care resources, especially in the administration of psychological interventions, a group setting delivery to a wider GI population may be cost effective. Specific alterations in gene expression, impacting key immune processes and inflammatory pathways linked to IBD, were induced by the RR-MBI, suggesting that an RR-MBI may elicit not only beneficial psychological effects, but also pathophysiological effects on inflammatory pathways directly involved in IBD manifestations.

## Supporting Information

S1 TREND Checklist TREND checklist. (PDF)

S1 Fig Interactive Network representation of the biological functions significantly altered by 8-weeks of RR-MBI among IBS patients. Networks shown: A) Cell cycle and cellular proliferation related genes with UBC, MAPK and TNF as primary regulatory nodes; B) Genes involved in hematological systems and inflammation with TP53 and NF-κB as a critical regulatory node. We used the Ingenuity Pathways Analysis tool (IPA 8.0) to generate the networks of genes altered by RR-MBI among **IBS** patients and merged the major networks with related functions. Each node represents a gene and each edge represent a molecular interaction. The intensity of the node color indicates the degree of upregulation (red) and downregulation (green), while white nodes indicate non-modified genes that may be affected in a non-transcriptional manner.
(TIF)

S2 Fig Enrichment map of pathways significantly affected by the RR-MBI among IBS patients. The significantly affected pathways were identified on the basis of normalized p value and False Discovery Rate using a Gene Set Enrichment Analysis (GSEA) approach. In the enrichment map, each node represents a biological pathway and each line represents a group of common enriched genes between the connected pathways. The red and blue color nodes indicate up- and down- regulated pathways, respectively. Thicker lines indicated a larger number of genes.
(PDF)

S3 Fig Enrichment map of pathways significantly affected by the RR-MBI among IBD patients. The significantly affected pathways were identified on the basis of normalized p value and False Discovery Rate using a Gene Set Enrichment Analysis (GSEA) approach. In the enrichment map, each node represents a biological pathway and each line represents a group of common enriched genes between the connected pathways. Blue color nodes indicate down- regulated pathways. In the IBD group, most pathways were downregulated after the RR-MBI. Thicker lines indicated a larger number of genes.
(PDF)

S1 Table Outcome measures after intervention and at 3-week follow-up for Irritable Bowel Syndrome (IBS) (N = 19, top) and Inflammatory Bowel Disease (IBD) (N = 29, bottom). (DOCX)

S2 Table List of Significantly differentially expressed genes between IBS and IBD at baseline. (XLSX)

S3 Table Genes commonly affected by 8-weeks of RR intervention in IBS and IBD groups. (DOCX)

S4 Table Gene-Ontology (GO) enrichment analysis of genes altered in IBS patients after the RR-MBI. The enrichment score for each biological group indicates its importance (enrichment) and was calculated by taking the geometric mean of enrichment p-values (EASE scores) for each GO term associated with the gene members in the group. An enrichment score of 2 is equivalent to a non-log p-value of 0.01.
(DOCX)

S1 Protocol Trial protocol. (DOC)
