# Correction: Genomic and Clinical Effects Associated with a Relaxation Response Mind-Body Intervention in Patients with Irritable Bowel Syndrome and Inflammatory Bowel Disease

**DOI:** 10.1371/journal.pone.0172872

**Published:** 2017-02-21

**Authors:** Braden Kuo, Manoj Bhasin, Jolene Jacquart, Matthew A. Scult, Lauren Slipp, Eric Isaac Kagan Riklin, Veronique Lepoutre, Nicole Comosa, Beth-Ann Norton, Allison Dassatti, Jessica Rosenblum, Andrea H. Thurler, Brian C. Surjanhata, Nicole N. Hasheminejad, Leslee Kagan, Ellen Slawsby, Sowmya R. Rao, Eric A. Macklin, Gregory L. Fricchione, Herbert Benson, Towia A. Libermann, Joshua Korzenik, John W. Denninger

There are errors in the Competing Interests statement. The correct Competing Interests statement is: The authors have the following conflicts to report: Dr. Kuo consults for Genova Diagnostics, Furiex, and Given Imaging. Dr. Kuo receives funding from Given and Furiex for clinical trials, unrelated to this particular study. Dr. Macklin serves on the DSMBs for Lantheus Medical Imaging, Shire Human Genetic Therapies, and Civitas Therapeutics. Dr. Denninger receives support for unrelated investigator-initiated studies from Onyx Pharmaceuticals and Basis. This does not alter the authors' adherence to PLOS ONE policies on sharing data and materials. The following authors hold or have held positions at the Benson-Henry Institute for Mind Body Medicine at Massachusetts General Hospital, which is paid by patients and their insurers for running the SMART-3RP and related relaxation/mindfulness clinical programs, markets related products such as books, DVDs, CDs and the like, and holds a patent pending (PCT/US2012/049539 filed August 3, 2012) entitled "Quantitative Genomics of the Relaxation Response": MB, JJ, MAS, LS, EIKR, VL, NNH, LK, ES, GLF, HB, TAL, JWD.
