# Supplementary figures and images for: Genomic and Clinical Effects Associated with a Relaxation Response Mind-Body Intervention in Patients with Irritable Bowel Syndrome and Inflammatory Bowel Disease
Source: PLoS One. 2015 Apr 30;10(4):e0123861. doi: 10.1371/journal.pone.0123861 (PMC4415769; doi:10.1371/journal.pone.0123861)

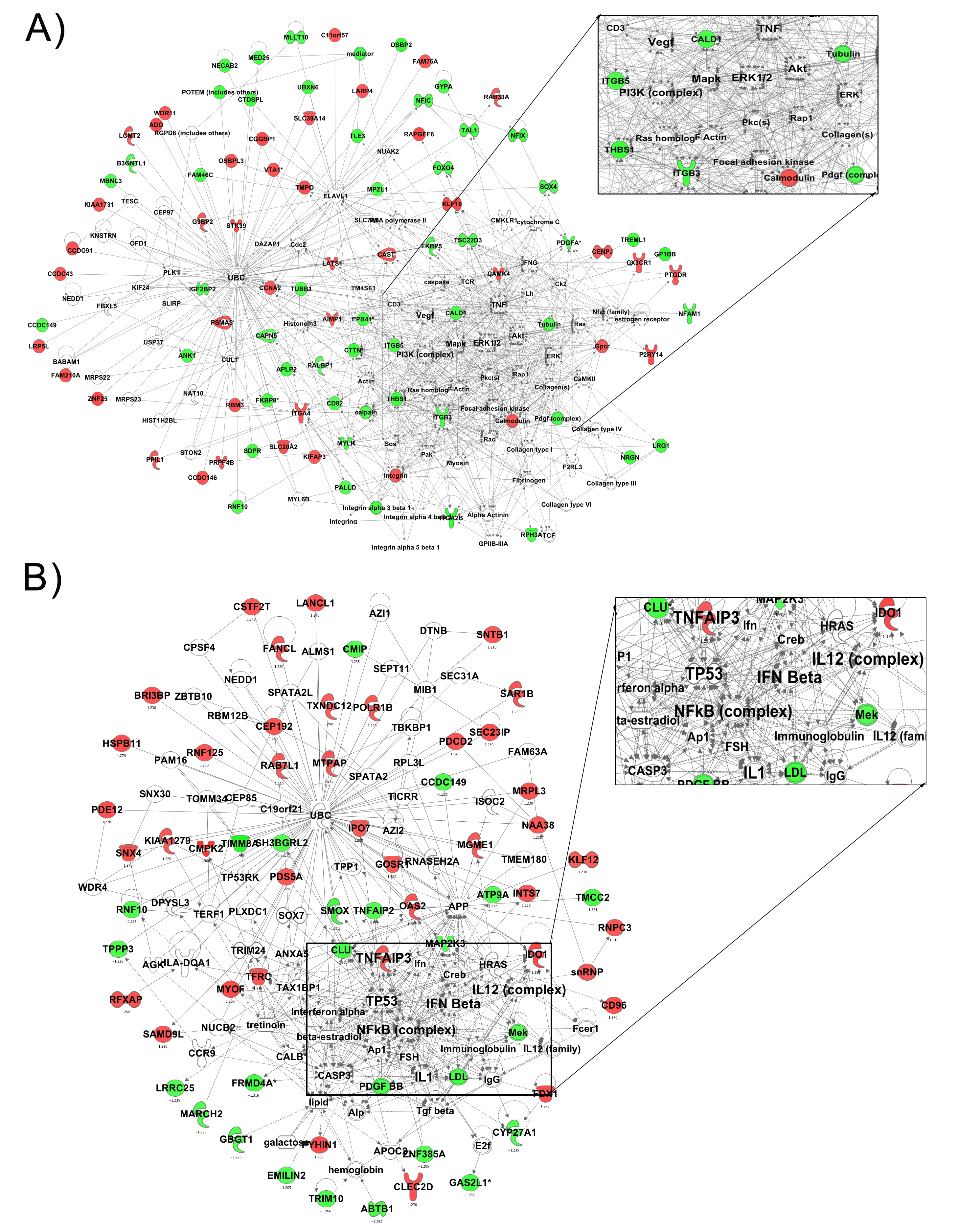

Supplement: S1 Fig — Networks shown: A) Cell cycle and cellular proliferation related genes with UBC, MAPK and TNF as primary regulatory nodes; B) Genes involved in hematological systems and inflammation with TP53 and NF-κB as a critical regulatory node. We used the Ingenuity Pathways Analysis tool (IPA 8.0) to generate the networks of genes altered by RR-MBI among IBS patients and merged the major networks with related functions. Each node represents a gene and each edge represent a molecular interaction. The intensity of the node color indicates the degree of upregulation (red) and downregulation (green), while white nodes indicate non-modified genes that may be affected in a non-transcriptional manner. (TIF) [file pone.0123861.s002.tif]

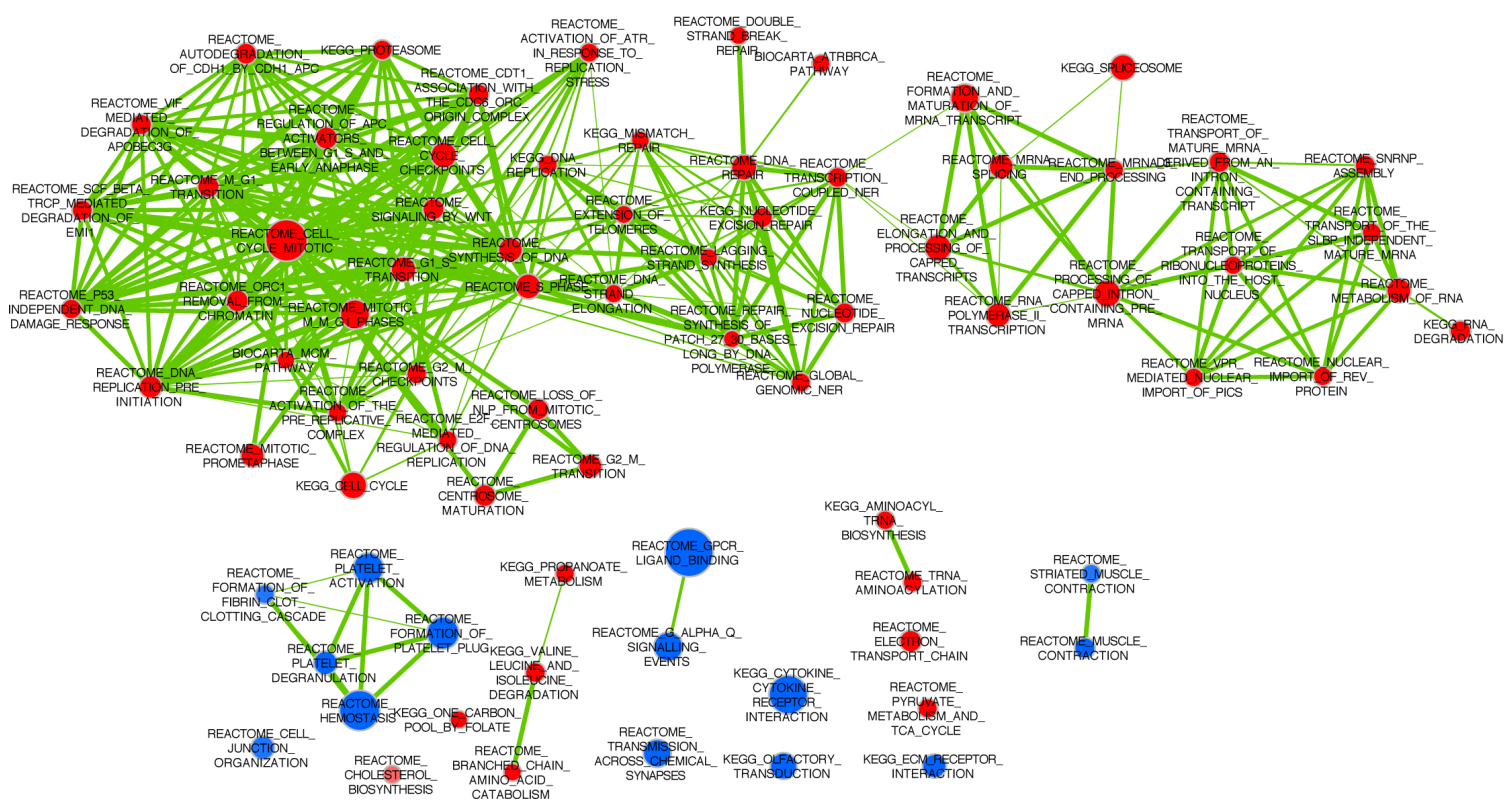

Supplement: S2 Fig — The significantly affected pathways were identified on the basis of normalized p value and False Discovery Rate using a Gene Set Enrichment Analysis (GSEA) approach. In the enrichment map, each node represents a biological pathway and each line represents a group of common enriched genes between the connected pathways. The red and blue color nodes indicate up- and down- regulated pathways, respectively. Thicker lines indicated a larger number of genes. (PDF) [file pone.0123861.s003.pdf]

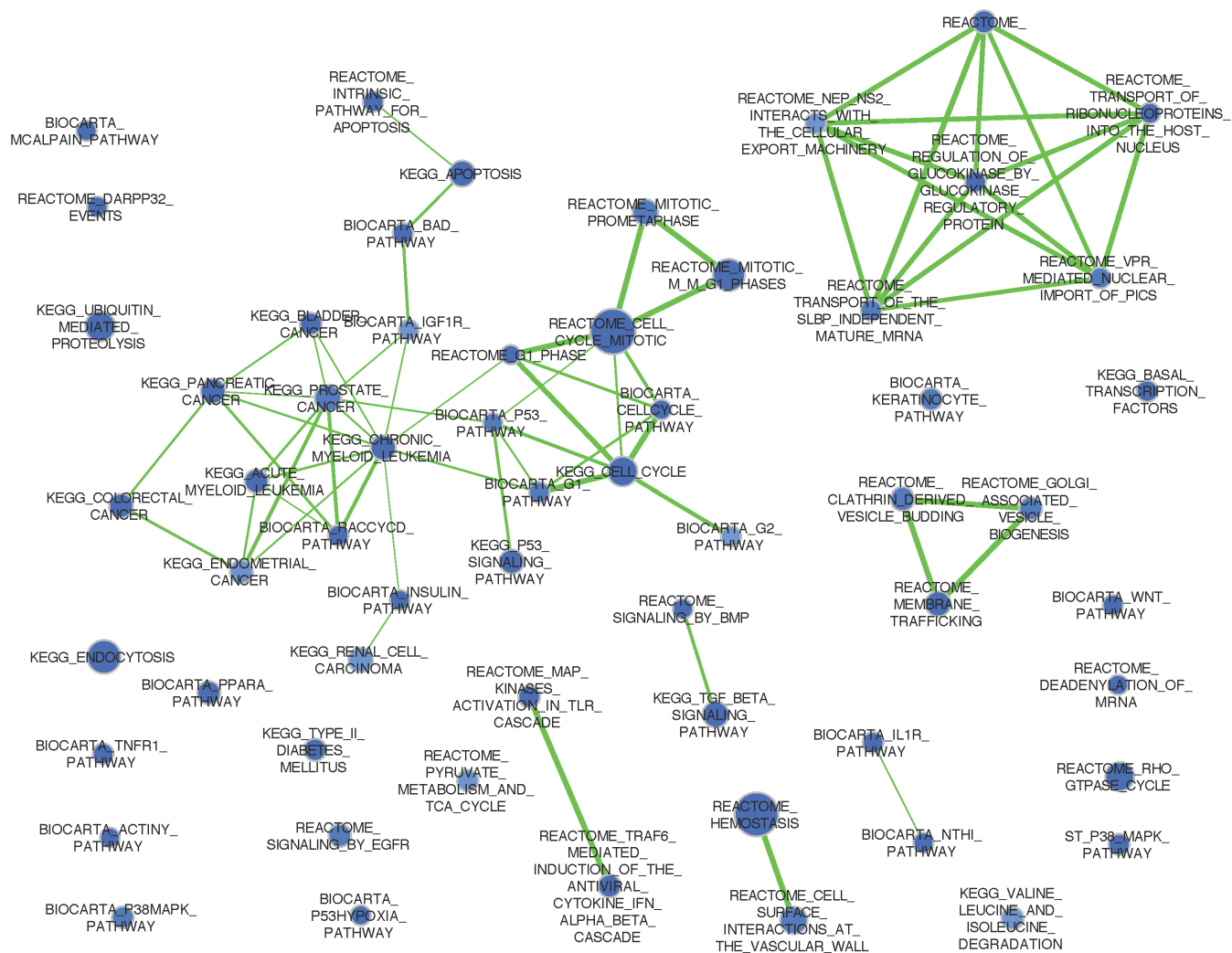

Supplement: S3 Fig — The significantly affected pathways were identified on the basis of normalized p value and False Discovery Rate using a Gene Set Enrichment Analysis (GSEA) approach. In the enrichment map, each node represents a biological pathway and each line represents a group of common enriched genes between the connected pathways. Blue color nodes indicate down- regulated pathways. In the IBD group, most pathways were downregulated after the RR-MBI. Thicker lines indicated a larger number of genes. (PDF) [file pone.0123861.s004.pdf]
