# Supplementary material for: Genomic and Clinical Effects Associated with a Relaxation Response Mind-Body Intervention in Patients with Irritable Bowel Syndrome and Inflammatory Bowel Disease
Source: PLoS One. 2015 Apr 30;10(4):e0123861. doi: 10.1371/journal.pone.0123861 (PMC4415769; doi:10.1371/journal.pone.0123861)
Supplement: S1 Table — (DOCX) [file pone.0123861.s005.docx]

**SUPPLEMENTAL TABLES**

| **Table S1.** Outcome measures after intervention and at 3-week follow-up for Irritable Bowel Syndrome (IBS) (N=19, top) and Inflammatory Bowel Disease (IBD) (N=29, bottom). | | | | | | | | | | | | | | | | |
| --- | --- | --- | --- | --- | --- | --- | --- | --- | --- | --- | --- | --- | --- | --- | --- | --- |
|  | Baseline  (Pre-Intervention) | | | | Week 5  (Mid-Intervention) | | | | Week 10  (Post-Intervention) | | | | Week 13  (Short-Term Follow-Up) | | | |
|  | IBS | | IBD | | IBS | | IBD | | IBS | | IBD | | IBS | | IBD | |
|  | Mean | SE | Mean | SE | Mean | SE | Mean | SE | Mean | SE | Mean | SE | Mean | SE | Mean | SE |
| IBD Questionnaire (IBD-Q) |  |  | 171.2 | 6.1 |  |  | 172.0. | 5.8 |  |  | 185.0** | 5.5 |  |  | 184.3* | 5.4 |
| IBS Quality of Life (IBS-QOL) | 67.1 | 5.1 |  |  | 71.5 | 4.3 |  |  | 74.8* | 3.5 |  |  | 80.6*** | 3.3 |  |  |
| IBS Symptom Severity Index (IBS-SSI) | 215.1 | 24.4 |  |  | 153.7** | 23.3 |  |  | 127.5*** | 23.5 |  |  | 147.1* | 26.6 |  |  |
| State-Trait Anxiety Inventory  (STAI-Y) – State Anxiety | 37.2 | 2.9 | 36.9 | 2.5 | 35.8 | 2.9 | 36.0 | 2.6 | 31.6 | 2.9 | 31.6 | 2.8 | 30.8 | 3.4 | 31.7 | 3.0 |
| State-Trait Anxiety Inventory  (STAI-Y) – Trait Anxiety | 39.0 | 2.8 | 39.3 | 2.4 | 35.9 | 2.7 | 36.6 | 2.3 | 34.3* | 2.5 | 32.4** | 2.3 | 33.7* | 2.6 | 33.6* | 2.5 |
| Pain Catastrophizing Scale (PCS) | 10.7 | 2.5 | 14.8 | 1.9 | 12.2 | 2.3 | 14.0 | 2.0 | 10.0 | 1.8 | 10.6* | 2.0 | 5.0* | 1.8 | 9.6** | 2.1 |
| Brief Pain Inventory (BPI) –  Pain Interference | 1.3 | 0.5 | 1.5 | 0.5 | 1.7 | 0.5 | 1.7 | 0.4 | 2.2 | 0.5 | 1.0 | 0.4 | 1.2 | 0.5 | 1.0 | 0.4 |
| Brief Pain Inventory (BPI) –  Pain Severity | 2.0 | 0.5 | 1.5 | 0.3 | 2.3 | 0.5 | 1.7 | 0.3 | 2.3 | 0.5 | 1.0 | 0.4 | 2.0 | 0.6 | 1.1 | 0.4 |
| Erythrocyte Sedimentation Rate (ESR) | 2.5 | 0.2 | 2.4 | 0.2 |  |  |  |  | 2.3 | 0.2 | 2.4 | 0.2 |  |  |  |  |
| C-reactive protein (CRP) | 0.4 | 0.3 | 0.9 | 0.2 |  |  |  |  | 0.6 | 0.3 | 0.8 | 0.3 |  |  |  |  |
| *P<0.05, **P<0.01, ***P<0.001 indicate mixed model analysis comparing baseline scores to post-intervention and short-term follow-up. | | | | | | | | | | | | | | | | |
