# Supplementary material for: Genomic and Clinical Effects Associated with a Relaxation Response Mind-Body Intervention in Patients with Irritable Bowel Syndrome and Inflammatory Bowel Disease
Source: PLoS One. 2015 Apr 30;10(4):e0123861. doi: 10.1371/journal.pone.0123861 (PMC4415769; doi:10.1371/journal.pone.0123861)
Supplement: S3 Table — (DOCX) [file pone.0123861.s007.docx]

**SUPPLEMENTAL TABLES**

| **Table S3**: Genes commonly affected by 8-weeks of RR intervention in IBS and IBD groups. | | | | | |
| --- | --- | --- | --- | --- | --- |
|  |  | **IBS** | | **IBD** | |
| **Symbol** | **Name** | **Ratio(Post/Pre)** | **P value** | **Ratio(Post/Pre)** | **P value** |
| CTTN | cortactin | 0.73 | 0.0065492 | 0.76 | 0.0076428 |
| FKBP5 | FK506 binding protein 5 | 0.76 | 0.0123404 | 0.72 | 0.023173 |
| CTDSPL | CTD (carboxy-terminal domain, RNA polymerase II, polypeptide A) small phosphatase-like | 0.76 | 0.0119947 | 0.83 | 0.0202512 |
| CTTN | cortactin | 0.72 | 0.0154611 | 0.68 | 0.0071394 |
| ITGB3 | integrin, beta 3 (platelet glycoprotein IIIa, antigen CD61) | 0.77 | 0.0133799 | 0.81 | 0.0017775 |
| FRMD4A | FERM domain containing 4A | 0.82 | 0.0225801 | 0.83 | 0.0103573 |
| SH3BGRL2 | SH3 domain binding glutamic acid-rich protein like 2 | 0.81 | 0.0244347 | 0.81 | 0.0170757 |
| TAL1 | T-cell acute lymphocytic leukemia 1 | 0.78 | 0.0239403 | 0.79 | 0.0046839 |
| OAS2 | 2'-5'-oligoadenylate synthetase 2, 69/71kDa | 1.25 | 0.0286242 | 1.26 | 0.0202044 |
| CLU | clusterin | 0.76 | 0.0309411 | 0.82 | 0.028086 |
| TRIM58 | tripartite motif containing 58 | 0.81 | 0.030843 | 0.79 | 0.04151 |
| SDPR | serum deprivation response | 0.76 | 0.035188 | 0.75 | 0.0096287 |
| TUBB1 | tubulin, beta 1 | 0.81 | 0.0472855 | 0.77 | 0.0332303 |
