# Supplementary material for: Genomic and Clinical Effects Associated with a Relaxation Response Mind-Body Intervention in Patients with Irritable Bowel Syndrome and Inflammatory Bowel Disease
Source: PLoS One. 2015 Apr 30;10(4):e0123861. doi: 10.1371/journal.pone.0123861 (PMC4415769; doi:10.1371/journal.pone.0123861)
Supplement: S4 Table — The enrichment score for each biological group indicates its importance (enrichment) and was calculated by taking the geometric mean of enrichment p-values (EASE scores) for each GO term associated with the gene members in the group. An enrichment score of 2 is equivalent to a non-log p-value of 0.01. (DOCX) [file pone.0123861.s008.docx]

**SUPPLEMENTAL TABLES**

| **Table S4:** Gene-Ontology (GO) enrichment analysis of genes altered in **IBS** patients after the RR-MBI. The enrichment score for each biological group indicates its importance (enrichment) and was calculated by taking the geometric mean of enrichment p-values (EASE scores) for each GO term associated with the gene members in the group. An enrichment score of 2 is equivalent to a non-log p-value of 0.01. | | | |
| --- | --- | --- | --- |
| **Major GO Cluster** | **Enrichment Score** | **Selected Processes** | **P value** |
| **Stress/inflammatory response** | 1.89 |  |  |
|  |  | - Cellular response to stress | 0.002 |
|  |  | - Inflammatory response | 0.020 |
| **Response to Vitamin** | 1.76 |  |  |
|  |  | - Response to vitamin A | 0.006 |
|  |  | - Response to Retinoic acid | 0.030 |
| **Protein transport** | 1.74 |  |  |
| **Hematopoiesis** | 1.47 |  |  |
|  |  | - Hematopoiesis | 0.020 |
|  |  | - Immune system process | 0.037 |
|  |  | - Cell activation | 0.047 |
| **Angiogenesis** | 1.41 |  |  |
|  |  | - Blood vessel morphogenesis | 0.043 |
|  |  | - Angiogenesis | 0.046 |
