# Supplementary material for: Genomic and Clinical Effects Associated with a Relaxation Response Mind-Body Intervention in Patients with Irritable Bowel Syndrome and Inflammatory Bowel Disease
Source: PLoS One. 2015 Apr 30;10(4):e0123861. doi: 10.1371/journal.pone.0123861 (PMC4415769; doi:10.1371/journal.pone.0123861)
Supplement: S1 Protocol — (DOC) [file pone.0123861.s009.doc]

**PARTNERS HUMAN RESEARCH COMMITTEE DETAILED PROTOCOL**

A pilot study on the efficacy of a 10-week mind body intervention on quality of life and inflammatory disease markers in patients with Irritable Bowel Syndrome (IBS) and Inflammatory Bowel Disease (IBD).

**I.** **BACKGROUND AND SIGNIFICANCE**

**Background and Significance —Irritable Bowel Syndrome**

Irritable bowel syndrome (IBS) is a group of functional bowel discomfort symptoms where pain or abdominal discomfort is associated with changes in bowel habits or defecation. The diagnosis of IBS is based on a series of symptoms criteria and investigations that have excluded organic disease. The challenge in defining IBS criteria has been the topic of much discussion over the years since it’s recognition as a prevalent complaint in primary care and gastroenterology clinics. Currently, the Rome lll criteria reflect the well documented research data on IBS and are accepted by most practitioners as the criteria used to diagnose this condition. [1]

Rome lll Criteria for the diagnosis of IBS (2006)

According to the Rome II committees and the Functional Brain Gut Research Group,[2] IBS can be diagnosed based on at least 12 weeks, which need not be consecutive, of the preceding 12 months there was abdominal discomfort or pain that had two out of three of these features:[3]

- Relieved with defecation; and/or
- Onset associated with a change in frequency of stool; and/or
- Onset associated with a change in form (appearance) of stool.

Symptoms that cumulatively support the diagnosis of IBS:

- Abnormal stool frequency (for research purposes, "abnormal" may be defined as greater than 3 bowel movements per day and less than 3 bowel movements per week);
- Abnormal stool form (lumpy/hard or loose/watery stool);
- Abnormal stool passage (straining, urgency, or feeling of incomplete evacuation);
- Bloating or feeling of abdominal distention.

Supportive symptoms of IBS:

- A) Fewer than three bowel movements a week
- B) More than three bowel movements a day
- C) Hard or lumpy stools
- D) Loose (mushy) or watery stools
- E) Straining during a bowel movement
- F) Urgency (having to rush to have a bowel movement)
- G) Feeling of incomplete bowel movement
- H) Passing mucus (white material) during a bowel movement
- I) Abdominal fullness, bloating, or swelling

**Diarrhea-predominant**: At least 1 of B, D, F and none of A, C, E; or at least 2 of B, D, F and one of A or E.
**Constipation-predominant**: At least 1 of A, C, E and none of B, D, F; or at least 2 of A, C, E and one of B, D, F.

The symptoms of IBS have been described by physicians starting in the 19^th^ century. Many of the current hypotheses for etiologies and pathophysiology hypotheses that were formulated and used as working hypotheses since the 1950s are still in existence today. These include: food intolerances, hormonal influences, post infectious, and hypersensitivity. While some providers clearly adhere to their own biases when confronted with a patient with IBS, current hypotheses for the etiology that are supported by research consist of motility disturbances, genetics, bacterial overgrowth, serotonin reuptake inhibitions, infection and inflammation, stress and food allergies. [4] In the evaluation of patients with IBS, psychosocial factors, stress sexual or physical abuse history is vital and requires an extra time commitment by the providers. There is typically a dominant symptom of either diarrhea (greater than 3 loose bowel movements/day or constipation (less than 3 bowel movements a week).

While the etiology of IBS continues to be sought, the diagnosis of
IBS is made by the identification of clinical symptoms consistent with the disorder when all organic causes have been ruled out. Treatment is dependent on the predominant symptom. In diarrhea-predominant symptoms, the use of anti diarrhea drugs and avoidance of certain food intolerances (lactose for those who are lactase deficient for example) and diarrhea inducing medications (for example serotonin reuptake inhibitors, SSRIs) are recommended. For patients with constipation-predominant symptoms the use of bulking agents (fiber), laxatives, avoidance of constipating diets (low fiber diets) and medications (narcotics) are recommended. Antispasmodics agents decrease intestinal smooth muscle active which may result in decreased of abdominal pain if this discomfort is secondary to smooth muscle spasms. The use of antidepressants is being used in concurrence with other interventions. These include SSRIs, and tricyclic antidepressants (TCAs) in lower doses that those used for antidepressant therapy.

Alternative therapies are currently in use either through provider recommendation or self referral by the patient. These include acupuncture, hypnotherapy and Chinese herbal medicine.

**Background and Significance – Inflammatory Bowel Disease**

IBD, comprising Crohn’s disease (CD) and ulcerative colitis (UC) are distinct from IBS in that characteristic chronic inflammation is identified in the small bowel and or colon. The two main diseases of IBD, UC and CD, may in themselves represent a heterogeneous set of diseases culminating on a common set of phenotypes. Both are chronic diseases with spontaneous flares and remissions of unclear etiology. Recent advances have identified genetic and some environmental risk factors which likely interact to provoke the diseases. UC involves confluent mucosal inflammation beginning in the rectum and spreading to various lengths of the colon (proctitis, proctosigmoiditis, left sided disease or pan-colitis). CD is characterized by inflammation essentially anywhere in the colon with the terminal ileum affected in over 70% of the time and the colon involved alone in 20%. Both diseases cause a core set of clinical symptoms which include diarrhea, abdominal pain or cramping and fatigue.

The role of stress in inducing the disease or provoking flares remains a source of controversy. Historically, UC was seen as a paradigm of a psychosomatic disease which leads to interventions as extreme as lobotomies.[5] Most patients remark that stress plays a central role in flares though this has been difficult to demonstrate in clinical studies. A Danish study assessed whether parents, without IBD, who had lost a child were at an increased risk of developing IBD and if parents with IBD who had lost a child were at increased risk of having a flare in the period after the child’s death. Neither endpoint was found to occur.[2] However, other studies have suggested that stress and depression does influence the course of UC or Crohn’s disease. Bitton et al [3]found that flares of UC were preceded more often by stressful events, though the effect was mild, but their study was small, following only 60 people one year and their confidence interval approached one (1.04). Levenstein et al [6] studied an Italian cohort of 62 UC patients for 68 months and showed an increased risk of exacerbation among those who had a higher level of perceived long-term stress (previous 2 years at baseline); though, a short term increase in perceived stress was not associated with an increase risk in flares. However, these findings were questionable because the study followed individuals for over 5 years and it seemed implausible to suggest that previous stress would influence flares at such an interval while short-term stress did not influence flares. In addition, they concluded that depressive symptoms, smoking, disease extent, and disease duration did not influence flares.

**Background and Significance - Mind Body Medicine**

IBS is currently accepted as being multifactor involving various factors including genetics influences. However, psychological factors have always been thought by most experts to play a vital role. As a result, there has been attention in the clinical setting. While there is a tendency by clinicians to classify an illness as psychological after all medical reasons have been exhausted, there is growing support of the idea of a disease that is both biological and psychosocial, integrating responses to both these elements of health. There has been extensive use of pharmacological agents in the treatment of IBS, and yet many patients experience no or abbreviated responses to this traditional approach. Thus, psychologically oriented treatments are currently being used by many practitioners. These approaches range from cognitive behavioral therapy (CBT), hypnotherapy, and psychotherapy, all of which have been tested with clinical trials. Reviews of these studies have been challenging because double blind studies are impossible given the nature of the interventions. Recently, two review articles published in 2008, evaluated the outcomes of behavioral therapies for IBS and show that hypnotherapy, psychotherapy and CBT have all shown efficacy in clinical trials. The benefits of these treatments are clear although the etiology remains uncertain [7].

*Mind Body Medicine and the Relaxation Response:* The “Relaxation Response” (RR) was first measured and described by Herbert Benson, MD, in the early 1970’s. The elicitation of the Relaxation Response “down-regulates” the sympathetic nervous system and is characterized by a set of measurable, predictable and reproducible physiologic changes that include reductions in VO_2_, VCO_2_, respiratory rate, blood pressure, and arterial blood lactate[8-17]. Additional physiologic characteristics are prominent low frequency heart rate oscillations [18, 19]and reduced responsively to plasma norepinephrine[20]. Although the precise mechanisms by which the RR induces these changes are not understood, it has been hypothesized that NO is generated resulting in vascular dilatation and decreased activation of the hypothalamic-pituitary-adrenal (HPA) axis[21].

Mind body interventions that include RR and resiliency factor enhancement have been reported to be useful therapeutically (often as an adjunct to medical treatment) in numerous conditions that are caused or exacerbated by stress [22] including: mild to moderate depression/anxiety[23]; anxiety[24-27]; headache[28-30]; back/neck pain[31]; myocardial ischemia[32]; premature ventricular contractions in stable ischemic heart disease [33]or hypertension[34-37]; osteoarthritis[31]; stress symptoms[38]; improved outcomes after cardiac and other surgery[39, 40]; pain relief and anxiety reduction in femoral arteriography and other invasive medical procedures[41, 42]; premenstrual syndrome[43]; infertility[44, 45]; psychosomatic complaints[46]; chronic pain[31, 47]; insomnia[48-50]; musculoskeletal disorders[51]; wound healing[52]; rheumatoid arthritis[53, 54]; fibromyalgia [55]and disease and treatment related symptoms of cancer[56-60]. In our recent review of the literature, we suggest that the RR may be an appropriate and relevant therapeutic state to counteract several stress-related disease processes[61].

*Mind Body Medicine and IBS/IBD:* Studies implicating certain factors (psychological and psychosocial stressors, heightened stress responsivity, immune abnormalities, distress-amplifying cognitive styles, psychiatric co morbidity, and nocioceptive abnormalities) in IBS have led to formulation of multi-level models which hypothesize a dynamic interaction between these factors. Mind body interventions intended to reduce stress may be a promising category of treatment for bowel disorders. Several studies support stress reduction techniques in the treatment of IBS. In a small study of RR meditation, individuals with IBS had a significant reduction in IBS symptom scores at 1 year follow-up when compared with baseline scores[62]. Blanchard et al., in a study of 16 patients, reported greater reduction in symptom scores for patients who underwent relaxation therapy as compared with a control group[63]. Multi-component interventions, combining relaxation therapy, education, and psychotherapy have also shown a reduction in gastrointestinal symptom scores, including continued improvement in symptoms at 4-year follow-up [64-68]. In a study by Elsenbruch et al., patients with ulcerative colitis in remission or those with low disease activity when treated with structured 60-hour training program over 10 weeks that included stress management training, moderate exercise, Mediterranean diet, behavioral techniques and self-care strategies showed significantly greater improvement in quality of life scores as measured by the SF-36 scale Mental Health and the Psychological Health Sum score when compared with changes observed in the usual-care waiting control group[69].

A recent paper by Lackner et al., explored how CBT worked for IBS by performing a SEM analysis of a randomized clinical trial [70]. CBT improved global IBS symptoms independent of its effect on distress, and these improvements were associated with improvements in QOL. Kearney et al’s 2008 article reviewed mind-body interventions for adults with IBS from articles found on MEDLINE. A hypnotherapy intervention showed improvements in GI symptoms, quality of life, disability, anxiety, depression and excess health-care costs. Studies suggest that patients with IBS benefit more from the generally accepted CBT treatment than routine medical care. Relaxation therapy or multimodal therapy (a combination of relaxation therapy, education and psychotherapy) is also suggested to be beneficial for treatment of IBS[71]. Blanchard et al performed a randomized study with three conditions: group-based cognitive therapy, psychoeducational support groups (active control), and intensive symptom and daily stress monitoring. The results showed that the CBT and psychoeducational groups were both superior in management of GI symptoms and patient global ratings to the symptom monitoring group, but there was no difference between the CBT and psychoeducational groups [72].

In summary, taking into account all the literature, we suggest that RR may be an appropriate and relevant therapeutic state to counteract several stress-related disease processes such as IBS and IBD[61]. This proposed study uses a well-developed mind body intervention (RR) that may provide symptom relief for IBS and IBD patients through the physiologic impact of the RR on biological parameters.

*Gene expression:* Though mind-body interventions had been occurring for many years, there had been little exploration of the impact of such interventions on gene expression until recently. A recent pair of studies, reported together,[73] first compared differential gene expression between long-time practitioners of mind-body techniques with matched controls who had never practiced these techniques (long-term practitioner study). Next, the authors then had the matched controls (previously naïve to mind-body techniques) undergo 8 weeks of RR training, and examined differential gene expression before and after the RR training (short-term practitioner study). The authors found, that from >40,000 genes evaluated, approximately 2000 genes were differentially expressed in both the long-term and short-term study, with 433 genes shared. In this cohort of genes, those genes associated with physiologic stress (e.g., oxidative stress) were overrepresented. Such findings, while provocative, require a prospective confirmation in a different and larger set of subjects.

**II**. **SPECIFIC AIMS**

The unique contribution of this study is the formal comparison of mind body interventions on two distinct gastrointestinal diseases—IBD and IBS. This study will assess the effect of a mind body group intervention on quality of life and inflammation in these two illnesses. Ultimately, such an intervention could provide a cost-effective means for easing the burden of IBS and IBD.

**Specific Aim 1:** To assess the effect of an open-label 10-week Mind Body group intervention on disease-specific and general quality of life measures on patients with IBS and IBD.

Hypothesis 1a: For IBS subjects, after completing the 10-week intervention, scores will improve on the Adequate Relief of IBS Pain (AR-IBS) and Subject Global Impression of Change (SGIC).

Improvement will be determined by the proportion of subjects who answer “yes” two out of three times on the AR-IBS at weeks 5, 10 and 13.

Improvement will be determined by the proportion of subjects who answer the three first choices (very much improved, much improved, minimally improved) using the SGIC at weeks 5, 10 and 13.

For IBS subjects, after completing 10 week intervention, scores will improve greater than 30% from baseline scores of discomfort relating to the Symptom Severity Index (SSI).

Hypothesis 1b: For IBD subjects, after completing the 10-week intervention, scores will improve on the IBDQ.

Improvement will be determined by the proportion of subjects whose score increases by > 15 points from baseline.

Hypothesis 1c: For both IBS and IBD subjects, after completing the 10-week intervention, scores will improve on the SF-36.

Improvement will be determined by the proportion of subjects that have statistically significant (p<0.05) improvements on SF 36 questionnaires.

**Specific Aim 2:** To assess the effect of an open-label 10-week Mind Body group intervention on response to the program either through self assessment or biological markers of inflammation.

Hypothesis 2a: For both IBS and IBD subjects, after completing the 10-week intervention, subjects will have a reduction of psychological and symptom distress.

This will be determined by the proportion of subjects that have significantly improved scores (p<0.05) on remaining self assessment questionnaires:

*IBS and IBD*

Modified Short Form Brief Pain Inventory (SF-BPI)

Pain Catastrophizing Scale (PCS)

Symptom Checklist-90-R (SCL-90-R)

Beck Depression Inventory II (BDI-II)

Spielberg State-Trait Anxiety Index (STAI-A)

*IBS Only*

Irritable Bowel Syndrome-Quality of Life Questionnaire (IBS-QOL)

Bristol Stool Scale (BSS) & IBS Subtype Criteria

Subject Impression of Change (SIC)

Symptom Severity Index (SSI) (symptom index modified to a 10-point scale)

Work Productivity and activity impairment questionnaire for Irritable Bowel Syndrome (WPAI-IBS)

EuroQOL Health Questionnaire (EQ-5D)

Stool Frequency Assessment

*IBD Only*

Harvey Bradshaw Index (Crohn’s patients only)

Simple Clinical Colitis Activity Index (SCCAI - Ulcerative Colitis patients only)

Hypothesis 2b: For both IBS and IBD subjects, after completing the 10-week intervention, subjects will have reduced systemic inflammation.

This will be determined by the proportion of subjects that have reduced erythrocyte sedimentation rate (ESR) and C-reactive protein (CRP) measurements.

Hypothesis 2c: For both IBS and IBD subjects, after completing the 10-week intervention, subjects will have significant changes in expression of the majority of the candidate genes (identified in our prior RR study of gene expression) thought to be responsive to mind body training.

This will be determined by the proportion of subjects that have significant changes in gene expression.

Hypothesis 2d: For both IBS and IBD subjects, after completing the 10-week intervention, subjects will have been symptom free for at least 5 weeks (not necessarily sequential) during the intervention phase.

**III.** **SUBJECT SELECTION**

30 patients (15 IBS/15 IBD) will be enrolled in the program ages 18 to 75. Subject recruitment will be done by the primary investigators and staff. Flyers will be placed in GI clinic offices with the permission of the clinic and recruitment letters will be sent out to health care providers. Advertisements will be sent to Partners’ employees and an advertisement will be published in the Metro newspaper. Identification of IBS and IBD patients who might benefit as seen by the clinicians will be another route of recruitment. Approach to participation will be education based, with an emphasis on the volunteer nature of participation. Patients will be approached if there is a potentially benefit identified for the patient with no regard of race, gender, or socioeconomic status.

**General Inclusion Criteria**

1. Subject is between the ages of 18 and 75
2. Male or female
3. Females of childbearing (reproductive) potential must have a negative serum pregnancy test at screening
4. Documentation of diagnosis by either primary care provider (PCP) or gastroenterologists (GI) will be accepted

**Inclusion criteria for IBS**

1. Subject has IBS confirmed by the Rome ll diagnostic criteria (see below) for 6 months

2. No changes in IBS medications for the last 4 weeks

3. HCG negative, no planned pregnancies for duration of the trial (24 weeks)

4. Subject must maintain a stable diet for the duration of the study

5. Subject is capable of understanding the requirement of the study, is willing to comply with all study procedures, understands the language of the informed consent and is capable and willing to sign the informed consent

**Inclusion Criteria for IBD**

1. Upper limit for HBI is 20; upper limit of SCCAI
2. Exclusion of NSAIDs
3. Stable medication for the previous 4 weeks

**General Exclusion Criteria**

1. Current sessions (< 3 weeks) with Tai Chi, meditation, yoga, individual mind/body based psychotherapy or counseling sessions
2. Psychotherapy initiated in the last 8 weeks
3. No concurrent total parental nutrition or tube feeding
4. Psychotropic medications (unless have been on stable psychotropic medications for at least 12 weeks)
5. Subject has current evidence of duodenal ulcer, gastric ulcer, diverticulitis, esophagitis or infectious gastroenteritis
6. Any acute gastrointestinal process
7. Subject has psychiatric disorder that is not controlled (based on investigators’ judgment)
8. Current use of steroids

**Exclusion Criteria for IBS**

1. H/o abdominal surgery in the past 5 years with the exception of appendectomy, cholycysectomy
2. H/o tube feeds or parenteral nutrition
3. Documentation of GI motility disorders

**Exclusion criteria for IBD**

1. Prednisone dose ≥ 20 mg
2. Change in medication anticipated in the next 10 weeks
3. Surgery anticipated in the next 10 weeks

**Prohibited Medications**

All concomitant medications taken during the study will be recorded in the case report form, along with dosage information and start and stop dates. Patients requiring excluded drugs will be discontinued from the study.

A total of 30 subjects will be recruited for this non-randomized pilot study, with a goal of 30 subjects (15 IBS and 15 IBD) completing the entire protocol. Patients will be recruited from the MGH Gastroenterology Clinic and from a vast network of primary care providers at MGH. Patients who show interest in participating in the study will talk to their physician while at the office visit or will call the MGH GI Clinic directly. When calling the clinic, patients will speak to the research nurse, who will ask screening questions based on the inclusion and exclusion criteria of the study. If the patient is potentially a candidate for the study, the nurse will schedule an appointment with the patient to come in for further information and an in-person screening visit.

**IV. SUBJECT ENROLLMENT**

##### Procedures for obtaining informed consent

**Initial evaluation/screening.** Upon arrival to GI Clinic, the patient will receive more information about the procedure and purpose of the study from their clinician. The patient will be screened again using inclusion and exclusion criteria (and medical history and physical exam to assess for medical exclusionary conditions). If the patient is eligible and continues to be interested in the study, s/he will be provided a full verbal explanation of the study and a written consent form.

**Consent procedure.** To ensure that subjects have the capacity to provide informed consent, we will ask potential enrollees to describe their understanding of the study’s purpose and their role (e.g., that they understand the structure and content of the visits and RR training; confidentiality and its limits; and their ability to end participation in the study at any time for any reason).

Subjects will be given adequate time (48 hours) to decide whether or not to enroll, and can ask questions about the study at the screening visit and after the visit. Subjects will also be reminded that participation in this study if voluntary.

##### Treatment assignment

All subjects will be assigned to active treatment for this non-randomized open treatment trial.

**V.** **STUDY PROCEDURES**

**Overview of Study Design**

This is a single center study which utilizes a mind body approach for the treatment of irritable bowel syndrome (IBS) and inflammatory bowel disease (IBD) using cognitive behavioral therapy, relaxation response techniques and meditation techniques as established by the Benson-Henry Institute for Mind Body Medicine (BHI) at Massachusetts General Hospital. Eligible subjects with either IBS or IBD will be enrolled as one cohort and undergo a 13-week group program facilitated by the BHI. All study subjects will enter the study (interventional stage) at the same time.

The study period is 13 weeks (91 days +/- 5 days). The study intervention period is 10 weeks with follow up visit at week 13. The estimated enrollment period is 4 months.

30 subjects will be recruited (15 IBS and 15 IBD).

**Study Design in Detail**

The pilot study will be performed in prospective cohort fashion with IBS and IBD patients. Once patients agree to participate in the study by signing the informed consent document, a full medical history will be taken and a physical examination will be performed.

**Pre-Interventional Phases**

**Pre-interventional Outline**

Screening by medical personnel

Baseline (3 weeks +/- 3 days before week one)

**Screening phase**

The screening phase will be the time period anytime before baseline. During the screening phase, patients will undergo a medical screening visit that will consist of a physical exam, medical history review and assessment for appropriateness in study participation and eligibility.

**Baseline Phase**

The first week treatment visit (week one of the intervention) will be the same for each subject as this is a group intervention. Given this study design the timing of the baseline visit in regards to the screening visit will vary with each patient.

The baseline phase will be the three week period of time leading up to the first treatment visit and will vary with each patient. The study will be conducted such that anytime within the 3 weeks (+/-3 days) prior to the first treatment visit, subjects will undergo a physical exam and eligibility parameters will be assessed, including biological markers. Thus, there will be subjects who screen in for the study, and undergo the baseline visit 2-4 months thereafter and there will be subjects who will screen in the for study and undergo the baseline visit with in a few days. For subjects whose screening visit is within 3 weeks for the interventional stage, the screening visit can be counted as the screening and baseline visit combined. At the baseline visit the subjects will fill out the second set of questionnaires which will record there symptoms and stress levels from the past two weeks. Thus, there is a chance of screen failures identified during this baseline period.

Safety note:

Study staff will review the patient's baseline surveys to screen for potential suicidality and homocidality. Potential suicidality and homocidality will be defined as a score of 2, 3 or 4 on Question 15 and Question 63 of the SCL-90-R. If a patient meets criteria for potential suicidality and/or homocidality, the study staff will immediately address the issue by assessing safety and follow standard procedures for patients demonstrating these traits during any study visits. In the event of a psychological emergency, confidentiality may be suspended. Participants will be informed of the limits of confidentiality at the beginning of the study.

**Interventional Phase**

Once recruitment has been complete and 30 subjects have been screened, a start date for the interventional phase will be established.

**Treatment phase**

The treatment phase will consists of weeks 1 through10, with a follow up visit at week 13. Eligible subjects with either IBS or IBD will placed in one cohort and undergo a 10 week program facilitated by the BHI Mind Body Institute. Each intervention session will last 1 hour and 30 minutes.

**Study visits in detail**

**Intervention**

Baseline: Physical exam, quality of life (QOL) questionnaires, blood draw for biological markers. Review of inclusion/exclusion criteria.

Week 1: Mind Body intervention session

Week 2: Mind Body intervention session

Week 3: Mind Body intervention session

Week 4: Mind Body intervention session

Week 5: Mind Body intervention session; QOL questionnaires

Week 6: Mind Body intervention session

Week 7: Mind Body intervention session

Week 8: Mind Body intervention session

Week 9: Mind Body intervention session

(End of Interventional phase)

Week 10: Mind Body intervention make-up session; QOL questionnaires and blood draw for biological markers

Week 12: no visit

Week 13: QOL questionnaires and physical exam

Screening questionnaires for health disease status, belief in intervention, stress, anxiety, and depression will be administered at screening baseline, week 5, 10 and 13. The Bristol Stool Scale (BSS) and IBS Subtype Criteria will be used for IBS patients to describe their stool and will be administered at baseline, weeks 5, 10 and 13. During the follow up period (week 13), the final self assessment will be made. Additionally, laboratories markers will be drawn and evaluated at baseline and week 10.

# IBS/IBD Syllabus

Session Content Homework

1. Introduction 1. Elicit RR Daily

Mind Body Medicine 2. Record Diary Sheet

Relaxation Response 3. Appreciation Journal

Teach RR (Autogenic)

2. Teach RR (Bath) 1. Elicit RR & Mini’s Daily

Discuss RR experiences from week 2. Record Diary Sheet

Diaphragmatic Breathing/Mini’s 3. Appreciation Journal

Teach New and Good/Positive Psychology 4. Pleasant Activity

Pleasant Activities

3. Teach RR (Body Scan/Beach Walk) 1. Elicit RR & Minis Daily

Review RR/Mini Practice 2. Record Diary Sheet

Review New and Good and Pleasant 3. Appreciation Journal

Activity 4. Activity Chart

Pacing/Activity Scheduling 5. Mindfulness

Mindfulness

4. Teach RR (Balloon) 1. Elicit RR a& Mini’s Daily

Review Pacing/Activity Scheduling & 2. Record Diary Sheet

Mindfulness 3. Cognitive Restructuring

Cognitive Restructuring (lemon)

Nutrition Lecture 45 min.

5. Teach RR (Magic Carpet) 1. Elicit RR & Mini’s Daily

Review Cognitive Restructuring 2. Record Diary Sheet

Cognitive Distortions/Goose In Bottle 3. Cognitive Restructuring

Cognitive Logs

Exercise Lecture 45 min.

6. Teach RR (Endorphins) 1. Elicit RR & Mini’s Daily

Physician Lecture and Q&A (1 Hour) 2. Record Diary Sheet

3. Cognitive Restructuring

7. Teach RR (Confronting Emotions) 1. Elicit RR & Mini’s Daily

Review Cognitive Restructuring 2. Record Diary Sheet

Journaling - Discuss Only 3. Journaling 3 days 20 min/day

8. Teach RR (Yoga 45 min.) 1. Elicit RR & Mini’s Daily

Review Journaling 2. Record Diary Sheet

Effective Problem Solving 3. Practice Comm. Skills

Communication Skills

9. Teach RR (Mountain) 1.Continue practice of all skills

Review Effective problem solving &

Communication Skills

Review Program Skills

10. Available for Make-ups if applicable

**Blood Draws for Biological Markers**

IBS and IBD group will look at erythrocyte sedimentation rate (ESR) and C-reactive protein (CRP) at baseline and week 10. 10ml of blood will be drawn for biomarkers ESR and CRP.

BHI group will draw 18.5 ml of blood into 5 PaxGene tubes (2.5ml in 4 tubes and 8.5ml in 1 tube) to look at gene expression at baseline and week 10.

*Gene expression analysis.* The transcriptional profile of samples will be probed using Affymetrix HG-U 133 Plus 2.0 chips according to previously described protocols[74] and prior work by our group.[73] Briefly, total RNA will be obtained from venous blood (2.5 ml) using the PAXgene Blood RNA System (Qiagen/BD); RNA purification, cDNA synthesis and *in vitro* transcription for production of biotin-labeled cRNA was performed using the CodeLink Expression Assay Reagent kit (GE Healthcare). Hybridization of cRNA with Affymetrix gene chips, and scanning of image output files will be performed according to previously described protocols.[74] Scanned image output files will be visually examined for major chip defects and hybridization artifacts and then analyzed with Affymetrix GeneChip Microarray Analysis Suite 5.0 (MAS5) software (Affymetrix). The image from each chip will be scaled such that the 2% trimmed mean intensity value for all arrays was adjusted to target intensity and reported as a non-negative quantity**.**

*Gene Ontology Analysis.* Genes that are differentially expressed between pre- and post-RR training will be analyzed using Expression Analysis Systematic Explorer (EASE) to identify biologically-relevant categories that are over-represented in the input set and therefore may be of further interest.[75] To accomplish this, EASE maps each probe to an Entrez Gene identifier,[76] which is associated with a gene ontology (GO) category:[77] biological function, cellular process, and molecular function. EASE identifies GO categories in the input gene list that are over-represented using jackknife iterative resampling of Fisher exact probabilities, with Bonferroni multiple testing correction. The "EASE score" is the upper bound of the distribution of Jackknife Fisher exact probabilities. Categories containing low numbers of genes are under-weighted so that the EASE score is more robust than the Fisher exact test. EASE analyses will test each list against all genes on the chip, and an EASE score will be calculated for likelihood of overrepresentation of a GO category in the input list. Overrepresentation describes a group of genes that belong to a certain GO category, e.g. cell cycle, that appear more often in the given input list than would be expected to occur if the distribution were random. The EASE score is a significance level with smaller EASE scores indicating increasing confidence in overrepresentation. We will select GO categories that have EASE scores of 0.05 or lower as significantly over-represented.

**Questionnaires and Scales**

**General Questionnaires**

- Short Form 36 (SF36): The SF-36 is a multi-purpose, short-form health survey with only 36 questions. It yields an 8-scale profile of functional health and well-being scores as well as psychometrically-based physical and mental health summary measures and a preference-based health utility index.
- Modified Short Form Brief Pain Inventory (SF-BPI): The SF-BPI is a validated, widely used, self-administered questionnaire developed to assess the severity of pain and the impact of pain on daily functions.
- Pain Catastrophizing Scale (PCS): The PCS is widely used to assess cognitive and affective responses to pain and to evaluate pain management program outcomes.
- Subject Global Impression of Change (SGIC): The SGIC consists of a self-evaluation by the patient of their overall change since the beginning of the study rated on a 7-point scale.
- SCL 90-R, Symptom Checklist-90-R: The SCL-90R evaluates a broad range of

psychological problems and symptoms of psychopathology. The instrument is also useful in measuring patient progress or treatment outcomes.

- Spielberg State-Trait Anxiety Index (STAI-Y): The STAI-Y is the definitive instrument for measuring anxiety in adults. It clearly differentiates between the temporary condition of “state anxiety” and the more general and long-standing quality of “trait anxiety”. It helps professionals distinguish between a client’s feelings of anxiety and depression.
- Beck Depression Inventory (BDI-II): The BDI–II consists of 21 items to assess the intensity of depression in clinical and normal patients. Each item is a list of four statements arranged in increasing severity about a particular symptom of depression.

**Irritable Bowel Syndrome Questionnaires**

- Irritable Bowel Syndrome-Quality of Life Questionnaire (IBS-QOL): The IBS-QOL is a self report quality of life measure containing 34 questions specific to IBS that is used to assess the impact of IBS and its treatment.
- Adequate relief of IBS pain (AR-IBS): This is a self-evaluation by the patient of their perception of adequate relief of IBS pain over the last 7 days as compared to before receiving treatment.
- Bristol Stool Scale (BSS) & IBS subtype Criteria: A scale that categorizes stool based on the description of its consistency. The instrument will be used to capture frequency and consistency of bowel movements.
- Subject Impression of Change (SIC): The SIC consists of a self-evaluation by the patient of their overall change since the beginning of the study rated on a 7-point scale.
- Symptom Severity Index (SSI) for bloating, discomfort, incomplete evacuation, straining, urgency: The SSI consists of a self-evaluation by the patient of their sense of bloating, discomfort, incomplete evacuation, straining and urgency rated on a 5-point scale.
- Work productivity and activity impairment questionnaire for Irritable Bowel Syndrome (WPAI-IBS): The WPAI questionnaire queries patients on how their IBS has impacted their ability to work (if employed) and do activities within the last 7 days.
- EuroQOL Health Questionnaire (EQ-5D): The EQ-5D is a general, single index measure for describing and valuing health-related quality of life. 5 dimensions that are measured are : mobility, self care, usual activities, pain/discomfort, and anxiety/depression. The instrument also includes a 20cm visual analog scale to capture health self-ratings.

**Inflammatory Bowel Disease Questionnaires**

- Quality of Life questionnaire (IBDQ): The IBDQ is designed to measure the effects of inflammatory bowel disease on daily function and quality of life.
- Harvey Bradshaw Index (Crohn’s Disease patients only): A validated clinical index used for Crohn's disease. It includes general well being, abdominal pain, number of liquid stools, abdominal mass and complications, and ranges from 0 to 25, with remission defined as a score below 5.
- Simple Clinical Colitis Activity Index (SCCAI-UC patients only): The SCCAI is a validated symptom based index (score 0-19) which has a good correlation with more complicated disease indices.

**VI.** **BIOSTATISTICAL ANALYSIS**

We will conduct exploratory univariate analyses on the relationship of the demographic and outcome variables. We will then complete a series of pre-post paired t-tests to estimate cumulated changes in the physical and psychological outcomes from baseline to follow-up. We will then conduct ANCOVA to examine the impact of demographic variables on the psychological and physical post-intervention outcomes, controlling for baseline psychological and physical outcomes scores.

**VII.** **RISKS AND DISCOMFORTS**

Responding to the listed questionnaires may provoke uncomfortable, negative emotions or cause mild stress. Subjects who experience any of these symptoms may choose to withdraw from the study.

There is also a risk to confidentiality. This is minimized by employing anonymous study IDs, limiting the number of people with access to the respective identifying information and storing all study related information and data in secure locations.

Some people may be uncomfortable with having their blood drawn and may feel lightheaded, dizzy or even faint. Other risks and discomforts associated with drawing blood from a vein include pain or bruising at the site of the blood draw and rarely, infection at this site.

A safety concern related to patients in this study is regarding suicidal ideation or homicidal thoughts. If patients express suicidal ideation (on the BDI-II (item #9), or a score of 2, 3 or 4 on Question 15 on the SCL-90-R) or homicidal thoughts (a score of 2, 3 or 4 on Question 63 of the SCL-90-R), a study staff member will immediately address the issue by assessing safety and follow standard procedures for patients demonstrating these traits during any study visits. In the event of a psychological emergency, confidentiality may be suspended. Participants will be informed of the limits of confidentiality at the beginning of the study.

**VIII.** **POTENTIAL BENEFITS**

Potential benefits include learning about and using comprehensive mind body techniques that may provide symptom relief for IBS and IBD patients and that may decrease the effects of stress in daily life.

Benefits to society include an increased understanding of how a mind body intervention effects stress-related disease processes such as IBS and IBD.

**IX. MONITORING AND QUALITY ASSURANCE**

We do not plan to employ a Data Safety Monitoring Board because potential risks to subjects are minimal.

Throughout the study subjects will be monitored for the occurrence of events defined as any undesirable experience or unanticipated benefit. Lack of effect of treatment is not considered an event. All adverse events will be recorded on an adverse event form. The Principle Investigator has the responsibility for reporting serious adverse events (death, life threatening, serious injury or permanent disability) to PHRC within 24-72 hours of notification.

A fully featured relational database located on a central server and networked to data entry and data analysis workstations will be used. Also, conventional data verification systems that are programmed to prevent logic errors and reduce incorrect out of range values are employed. Periodic analysis of each data field is conducted by the study PI to examine the expected distributions of the data and to identify outliers for possible data collection or entry errors.

A unique anonymous indentifier will be assigned to each subject and all questionnaire data collected subsequently will be associated exclusively with this identifier. Clinical data, collected routinely in the Mind Body program, will also be coded and stored in research files (in addition to usual clinical charts).

All research related subject data will be entered to secure, password protected computers. Hard copies of completed questionnaires and other study materials (with coded subject identification) will be stored in locked files at the and Benson-Henry Institute for Mind Body Medicine at MGH. Only research staff and the study sponsor will have access to these locations.

**X.** **REFERENCES**

1. Podovei, M. and B. Kuo, *Irritable bowel syndrome: a practical review.* South Med J, 2006. **99**(11): p. 1235-42; quiz 1243-4, 1284.

2. Li, J., et al., *Psychological stress and inflammatory bowel disease: a follow-up study in parents who lost a child in Denmark.* Am J Gastroenterol, 2004. **99**(6): p. 1129-33.

3. Bitton, A., et al., *Psychosocial determinants of relapse in ulcerative colitis: a longitudinal study.* Am J Gastroenterol, 2003. **98**(10): p. 2203-8.

4. Lembo, A., V.Z. Ameen, and D.A. Drossman, *Irritable bowel syndrome: toward an understanding of severity.* Clin Gastroenterol Hepatol, 2005. **3**(8): p. 717-25.

5. Levy R, W.H., Herrmann J, et al, *Experiences with prefrontal lobotomy for intractable ulerative colitis; preliminary report.* JAMA, 1956. **160**: p. 1277-1280.

6. Levenstein, S., et al., *Stress and exacerbation in ulcerative colitis: a prospective study of patients enrolled in remission.* Am J Gastroenterol, 2000. **95**(5): p. 1213-20.

7. Whorwell, P.J., *Behavioral therapy for IBS.* Nat Clin Pract Gastroenterol Hepatol, 2009. **6**(3): p. 148-9.

8. Benson, H., et al., *Continuous measurement of O2 consumption and CO2 elimination during a wakeful hypometabolic state.* J Human Stress, 1975. **1**(1): p. 37-44.

9. Benson, H., *The relaxation response*. 1975, New York: William Morrow.

10. Greenwood, M.M. and H. Benson, *The efficacy of progressive relaxation in systematic desensitization and a proposal for an alternative competitive response--the relaxation response.* Behav Res Ther, 1977. **15**(4): p. 337-43.

11. Benson, H., T. Dryer, and L.H. Hartley, *Decreased VO2 consumption during exercise with elicitation of the relaxation response.* J Human Stress, 1978. **4**(2): p. 38-42.

12. Benson, H. and I.L. Goodale, *The relaxation response: your inborn capacity to counteract the harmful effects of stress.* J Fla Med Assoc, 1981. **68**(4): p. 265-7.

13. Benson, H., E. Stuart, and R. Friedman, *Cognitive therapy for hypertension.* Ann Intern Med, 1994. **120**(1): p. 91.

14. Peters, R.K., H. Benson, and J.M. Peters, *Daily relaxation response breaks in a working population: II. Effects on blood pressure.* Am J Public Health, 1977. **67**(10): p. 954-9.

15. Peng, C.K., et al., *Heart rate dynamics during three forms of meditation.* Int J Cardiol, 2004. **95**(1): p. 19-27.

16. Peng, C.K., et al., *Exaggerated heart rate oscillations during two meditation techniques.* Int J Cardiol, 1999. **70**(2): p. 101-7.

17. Hoffman, J.W., et al., *Reduced sympathetic nervous system responsivity associated with the relaxation response.* Science, 1982. **215**(4529): p. 190-2.

18. Cannon, W., *Bodily changes in pain, hunger, fear and rage; an account of recent research into the function of emotional excitement*. 1915, New York: Appleton and company.

19. Stefano, G.B., et al., *Basal nitric oxide limits immune, nervous and cardiovascular excitation: human endothelia express a mu opiate receptor.* Prog Neurobiol, 2000. **60**(6): p. 513-30.

20. Sterling, P.a.J.E., *Allostasis: A new paradigm to explain arousal pathology*. Handbook of Life Stress, cognition and health, ed. E.S.F.a.J. Reason. 1988, New York: J. Wiley and Sons.

21. McEwen, B.S., *Protective and damaging effects of stress mediators.* N Engl J Med, 1998. **338**(3): p. 171-9.

22. Sternberg, E.a.P.W.G., *The mind-body interaction in disease.* Scientific American, 1997. **7**(1): p. 8-15.

23. Kutz, I., et al., *Meditation as an adjunct to psychotherapy. An outcome study.* Psychother Psychosom, 1985. **43**(4): p. 209-18.

24. Miller, J.J., K. Fletcher, and J. Kabat-Zinn, *Three-year follow-up and clinical implications of a mindfulness meditation-based stress reduction intervention in the treatment of anxiety disorders.* Gen Hosp Psychiatry, 1995. **17**(3): p. 192-200.

25. Kabat-Zinn, J., et al., *Effectiveness of a meditation-based stress reduction program in the treatment of anxiety disorders.* Am J Psychiatry, 1992. **149**(7): p. 936-43.

26. Benson, H., et al., *Treatment of anxiety: a comparison of the usefulness of self-hypnosis and a meditational relaxation technique. An overview.* Psychother Psychosom, 1978. **30**(3-4): p. 229-42.

27. Nakao, M., et al., *Anxiety is a good indicator for somatic symptom reduction through behavioral medicine intervention in a mind/body medicine clinic.* Psychother Psychosom, 2001. **70**(1): p. 50-7.

28. Benson, H., B.P. Malvea, and J.R. Graham, *Physiologic correlates of meditation and their clinical effects in headache: an ongoing investigation.* Headache, 1973. **13**(1): p. 23-4.

29. Benson, H., H.P. Klemchuk, and J.R. Graham, *The usefulness of the relaxation response in the therapy of headache.* Headache, 1974. **14**(1): p. 49-52.

30. Fentress, D.W., et al., *Biofeedback and relaxation-response training in the treatment of pediatric migraine.* Dev Med Child Neurol, 1986. **28**(2): p. 139-46.

31. Astin, J.A., *Mind-body therapies for the management of pain.* Clin J Pain, 2004. **20**(1): p. 27-32.

32. Blumenthal, J.A., et al., *Stress management and exercise training in cardiac patients with myocardial ischemia. Effects on prognosis and evaluation of mechanisms.* Arch Intern Med, 1997. **157**(19): p. 2213-23.

33. Benson, H., S. Alexander, and C.L. Feldman, *Decreased premature ventricular contractions through use of the relaxation response in patients with stable ischaemic heart-disease.* Lancet, 1975. **2**(7931): p. 380-2.

34. Benson, H., et al., *Decreased blood-pressure in pharmacologically treated hypertensive patients who regularly elicited the relaxation response.* Lancet, 1974. **1**(7852): p. 289-91.

35. Benson, H., et al., *Decreased blood pressure in borderline hypertensive subjects who practiced meditation.* J Chronic Dis, 1974. **27**(3): p. 163-9.

36. Stuart, E.M., et al., *Nonpharmacologic treatment of hypertension: a multiple-risk-factor approach.* J Cardiovasc Nurs, 1987. **1**(4): p. 1-14.

37. Leserman, J.e.a., *Nonpharmacologic intervention for hypertension: Long term follow up.* J Cardiopulmonary Rehabil, 1989. **9**: p. 316-24.

38. Himelhoch, S., D.R. Medoff, and G. Oyeniyi, *Efficacy of group psychotherapy to reduce depressive symptoms among HIV-infected individuals: a systematic review and meta-analysis.* AIDS Patient Care STDS, 2007. **21**(10): p. 732-9.

39. Domar, A.D., J.M. Noe, and H. Benson, *The preoperative use of the relaxation response with ambulatory surgery patients.* Hosp Top, 1987. **65**(4): p. 30-5.

40. Leserman, J., et al., *The efficacy of the relaxation response in preparing for cardiac surgery.* Behav Med, 1989. **15**(3): p. 111-7.

41. Mandle, C.L., et al., *Relaxation response in femoral angiography.* Radiology, 1990. **174**(3 Pt 1): p. 737-9.

42. Lang, E.V., et al., *Adjunctive non-pharmacological analgesia for invasive medical procedures: a randomised trial.* Lancet, 2000. **355**(9214): p. 1486-90.

43. Goodale, I.L., A.D. Domar, and H. Benson, *Alleviation of premenstrual syndrome symptoms with the relaxation response.* Obstet Gynecol, 1990. **75**(4): p. 649-55.

44. Domar, A.D., M.M. Seibel, and H. Benson, *The mind/body program for infertility: a new behavioral treatment approach for women with infertility.* Fertil Steril, 1990. **53**(2): p. 246-9.

45. Domar, A.D., et al., *Psychological improvement in infertile women after behavioral treatment: a replication.* Fertil Steril, 1992. **58**(1): p. 144-7.

46. Hellman, C.J., et al., *A study of the effectiveness of two group behavioral medicine interventions for patients with psychosomatic complaints.* Behav Med, 1990. **16**(4): p. 165-73.

47. Caudill, M., et al., *Decreased clinic utilization by chronic pain patients after behavioral medicine intervention.* Pain, 1991. **45**(3): p. 334-5.

48. Jacobs, G.D., H. Benson, and R. Friedman, (1993) *Home-based central nervous system assessment of multifactor behavioral intervention for chronic sleep-onset insomnia*. Behav Ther **Volume**, 159-74

49. Jacobs, G.D., et al., *Multifactor behavioral treatment of chronic sleep-onset insomnia using stimulus control and the relaxation response. A preliminary study.* Behav Modif, 1993. **17**(4): p. 498-509.

50. Morin, C.M., J.P. Culbert, and S.M. Schwartz, *Nonpharmacological interventions for insomnia: a meta-analysis of treatment efficacy.* Am J Psychiatry, 1994. **151**(8): p. 1172-80.

51. Luskin, F.M., et al., *A review of mind/body therapies in the treatment of musculoskeletal disorders with implications for the elderly.* Altern Ther Health Med, 2000. **6**(2): p. 46-56.

52. Wientjes, K.A., *Mind-body techniques in wound healing.* Ostomy Wound Manage, 2002. **48**(11): p. 62-7.

53. Astin, J.A., et al., *Psychological interventions for rheumatoid arthritis: a meta-analysis of randomized controlled trials.* Arthritis Rheum, 2002. **47**(3): p. 291-302.

54. Broderick, J.E., *Mind-body medicine in rheumatologic disease.* Rheum Dis Clin North Am, 2000. **26**(1): p. 161-76, xi.

55. Astin, J.A., et al., *The efficacy of mindfulness meditation plus Qigong movement therapy in the treatment of fibromyalgia: a randomized controlled trial.* J Rheumatol, 2003. **30**(10): p. 2257-62.

56. Syrjala, K.L., et al., *Relaxation and imagery and cognitive-behavioral training reduce pain during cancer treatment: a controlled clinical trial.* Pain, 1995. **63**(2): p. 189-98.

57. Burish, T.G. and J.N. Lyles, *Effectiveness of relaxation training in reducing adverse reactions to cancer chemotherapy.* J Behav Med, 1981. **4**(1): p. 65-78.

58. Spiegel, D. and R. Moore, *Imagery and hypnosis in the treatment of cancer patients.* Oncology (Williston Park), 1997. **11**(8): p. 1179-89; discussion 1189-95.

59. Syrjala, K.L., C. Cummings, and G.W. Donaldson, *Hypnosis or cognitive behavioral training for the reduction of pain and nausea during cancer treatment: a controlled clinical trial.* Pain, 1992. **48**(2): p. 137-46.

60. Vasterling, J., et al., *Cognitive distraction and relaxation training for the control of side effects due to cancer chemotherapy.* J Behav Med, 1993. **16**(1): p. 65-80.

61. Esch, T., et al., *Stress-related diseases -- a potential role for nitric oxide.* Med Sci Monit, 2002. **8**(6): p. RA103-18.

62. Keefer, L. and E.B. Blanchard, *A one year follow-up of relaxation response meditation as a treatment for irritable bowel syndrome.* Behav Res Ther, 2002. **40**(5): p. 541-6.

63. Blanchard, E.B., et al., *Relaxation training as a treatment for irritable bowel syndrome.* Biofeedback Self Regul, 1993. **18**(3): p. 125-32.

64. Schwarz, S.P., E.B. Blanchard, and D.F. Neff, *Behavioral treatment of irritable bowel syndrome: a 1-year follow-up study.* Biofeedback Self Regul, 1986. **11**(3): p. 189-98.

65. Blanchard, E.B.e.a., *A multi-component treatment for irritable bowel syndrome.* Behav Ther, 1988. **19**: p. 67-73.

66. Neff, D.F.a.B.E.B., *A multi-component treatment for irritable bowel syndrome.* Behav Ther, 1987. **18**: p. 70-83.

67. Guthrie, E., et al., *A controlled trial of psychological treatment for the irritable bowel syndrome.* Gastroenterology, 1991. **100**(2): p. 450-7.

68. Schwarz, S.P., et al., *Behaviorally treated irritable bowel syndrome patients: a four-year follow-up.* Behav Res Ther, 1990. **28**(4): p. 331-5.

69. Elsenbruch, S., et al., *Effects of mind-body therapy on quality of life and neuroendocrine and cellular immune functions in patients with ulcerative colitis.* Psychother Psychosom, 2005. **74**(5): p. 277-87.

70. Lackner, J.M., et al., *How does cognitive behavior therapy for irritable bowel syndrome work? A mediational analysis of a randomized clinical trial.* Gastroenterology, 2007. **133**(2): p. 433-44.

71. Kearney, D.J. and J. Brown-Chang, *Complementary and alternative medicine for IBS in adults: mind-body interventions.* Nat Clin Pract Gastroenterol Hepatol, 2008. **5**(11): p. 624-36.

72. Blanchard, E.B., et al., *A controlled evaluation of group cognitive therapy in the treatment of irritable bowel syndrome.* Behav Res Ther, 2007. **45**(4): p. 633-48.

73. Dusek, J.A., et al., *Genomic counter-stress changes induced by the relaxation response.* PLoS ONE, 2008. **3**(7): p. e2576.

74. Jones, J., et al., *Gene signatures of progression and metastasis in renal cell cancer.* Clin Cancer Res, 2005. **11**(16): p. 5730-9.

75. Hosack, D.A., et al., *Identifying biological themes within lists of genes with EASE.* Genome Biol, 2003. **4**(10): p. R70.

76. Maglott, D., et al., *Entrez Gene: gene-centered information at NCBI.* Nucleic Acids Res, 2005. **33**(Database issue): p. D54-8.

77. Ashburner, M., et al., *Gene ontology: tool for the unification of biology. The Gene Ontology Consortium.* Nat Genet, 2000. **25**(1): p. 25-9.
